# The Broad-Spectrum Antimicrobial Potential of [Mn(CO)_4_(S_2_CNMe(CH_2_CO_2_H))], a Water-Soluble CO-Releasing Molecule (CORM-401): Intracellular Accumulation, Transcriptomic and Statistical Analyses, and Membrane Polarization

**DOI:** 10.1089/ars.2017.7239

**Published:** 2018-05-10

**Authors:** Lauren K. Wareham, Samantha McLean, Ronald Begg, Namrata Rana, Salar Ali, John J. Kendall, Guido Sanguinetti, Brian E. Mann, Robert K. Poole

**Affiliations:** ^1^ Department of Molecular Biology and Biotechnology, The University of Sheffield, Sheffield, United Kingdom.; ^2^ School of Science and Technology, Nottingham Trent University, Nottingham, United Kingdom.; ^3^ School of Informatics, The University of Edinburgh, Edinburgh, United Kingdom.; ^4^ Department of Chemistry, The University of Sheffield, Sheffield, United Kingdom.

**Keywords:** antibacterial agents, carbon monoxide-releasing molecule, manganese carbonyl compound, systems biology, uncoupling agent

## Abstract

***Aims:*** Carbon monoxide (CO)-releasing molecules (CORMs) are candidates for animal and antimicrobial therapeutics. We aimed to probe the antimicrobial potential of a novel manganese CORM.

***Results:*** [Mn(CO)_4_S_2_CNMe(CH_2_CO_2_H)], CORM-401, inhibits growth of *Escherichia coli* and several antibiotic-resistant clinical pathogens. CORM-401 releases CO that binds oxidases *in vivo*, but is an ineffective respiratory inhibitor. Extensive CORM accumulation (assayed as intracellular manganese) accompanies antimicrobial activity. CORM-401 stimulates respiration, polarizes the cytoplasmic membrane in an uncoupler-like manner, and elicits loss of intracellular potassium and zinc. Transcriptomics and mathematical modeling of transcription factor activities reveal a multifaceted response characterized by elevated expression of genes encoding potassium uptake, efflux pumps, and envelope stress responses. Regulators implicated in stress responses (CpxR), respiration (Arc, Fnr), methionine biosynthesis (MetJ), and iron homeostasis (Fur) are significantly disturbed. Although CORM-401 reduces bacterial growth in combination with cefotaxime and trimethoprim, fractional inhibition studies reveal no interaction.

***Innovation:*** We present the most detailed microbiological analysis yet of a CORM that is not a ruthenium carbonyl. We demonstrate CO-independent striking effects on the bacterial membrane and global transcriptomic responses.

***Conclusions:*** CORM-401, contrary to our expectations of a CO delivery vehicle, does not inhibit respiration. It accumulates in the cytoplasm, acts like an uncoupler in disrupting cytoplasmic ion balance, and triggers multiple effects, including osmotic stress and futile respiration.

***Rebound Track:*** This work was rejected during standard peer review and rescued by rebound peer review (*Antioxid Redox Signal* 16: 293–296, 2012) with the following serving as open reviewers: Miguel Aon, Giancarlo Biagini, James Imlay, and Nigel Robinson. *Antioxid. Redox Signal.* 28, 1286–1308.

## Introduction

There is an urgent need for new antimicrobial agents; carbon monoxide (CO)—a poisonous gas that avidly binds to ferrous hemes in globins and oxidases inhibiting respiration (29)—may, in principle, be a potent antimicrobial molecule. However, CO also plays essential physiological roles (35) as a gasotransmitter (50) [or small-molecule signaling agent (18)]. CO is endogenously produced by heme oxygenase that catalyzes the degradation of heme, liberating CO, which then modulates key anti-inflammatory, antiapoptotic, and cytoprotective effects. However, the handling and health risks associated with administering CO gas have prompted the design and administration of CO-releasing molecules (CORMs), predominantly metal carbonyl compounds, allowing substantial advances in the biochemistry and physiology of CO (8, 37). Diverse CORMs differ in structure, kinetics, and in CO release mechanisms (70).

Innovation It is essential to investigate new carbon monoxide-releasing molecules (CORMs) if the promised prospects of site-specific and time-controlled release of CO are to be exploited. We report the first detailed microbiological characterization of the toxicity of the water-soluble CORM-401, [Mn(CO)_4_(S_2_CNMe(CH_2_CO_2_H))], to *Escherichia coli* strains and other pathogens. Our findings that CORM-401 is an ineffective inhibitor of growth and respiration (despite being accumulated to high levels), yet exerts profound effects on the bacterial membrane and global gene expression, cast doubt on the mechanism of action of this CORM and others. Such insights open the way for new compound design and novel, clinical combinatorial therapies.

The first water-soluble CORM to be synthesized and used biologically—[RuCl(glycinate)(CO)_3_], CORM-3—has been widely exploited in models of vascular dysfunction, inflammation, and ischemic injury (60). Subsequently, CORM-3 and other CORMs have been evaluated as antibacterial agents that target critical oxidases or other iron sites, that is, targets distinct from those of established antibiotics (9, 38). CORM-3 remains the best studied CORM, although with a complex solution chemistry (39), and over 150 articles have appeared on its actions since 2003.

Rigorous bacterial chemostat experiments involving transcriptomic datasets and mathematical modeling have revealed unexpected aspects of CORM-3 biochemistry. First, not only respiratory function but also diverse biological processes are affected by CORM-3(38). Second, CORM-2 and CORM-3 elicit effects that cannot be mimicked by CO gas, even at concentrations much higher than the ruthenium (Ru) CORMs (73). Third, even CO-depleted control molecules (so-called inactive CORM-3, iCORM) modulate bacterial gene expression, despite the inability to detect significant CO release from such molecules in the myoglobin assay (38). Finally, although heme is the classical target of CO in biological chemistry, heme-deficient bacteria are also sensitive to CORM-3 and display complex patterns of gene expression in response to this compound (74).

In view of the potential importance of CORMs as antibacterial agents and the mounting evidence for involvement of the metal and co-ligand fragment of CORMs in their biological effects (38, 74), we have used a newer compound that lacks Ru, a biologically foreign molecule. [Mn(CO)_4_S_2_CNMe(CH_2_CO_2_H)], CORM-401, is a CO-releasing manganese complex providing up to 3.2 moles CO per mole of compound (7), depending upon the concentration of the CO acceptor myoglobin and the presence of oxidants (16). The mechanism of CO loss is dissociative and reversible; reversible binding of CO results in a relatively stable solution of the compound (7).

Rebound Track This work was rejected during standard peer review and rescued by rebound peer review (*Antioxid Redox Signal* 16: 293–296, 2012) with the following serving as open reviewers: Miguel Aon, Giancarlo Biagini, James Imlay, and Nigel Robinson. The comments by these reviewers supporting the rescue are listed below:

**Miguel Aon** (*miguel.aon@nih.gov*): I am a qualified reviewer (per *Antioxid Redox Signal* 16: 293–296) and move to rescue this article that was rejected during the regular peer review process after reviewing all versions of the article and detailed reviewer comments. The article entitled *The Broad-Spectrum Antimicrobial Potential of [Mn(CO)_4_(S_2_CNMe(CH_2_CO_2_H))], a Water-Soluble CO-Releasing Molecule (CORM-401): Intracellular Accumulation, Transcriptomic and Statistical Analyses, and Membrane Polarization* represents a comprehensive in-depth assessment of the antibiotic function of CORM-401, a carbon monoxide-releasing manganese-based compound. The most important scientific contribution of this work is the detailed assessment of the mechanism of action of CORM-401 to inhibit the growth of bacteria. The emerging picture is of a pleiotropic nature, with multiple targets that functionally converge on adverse bioenergetic effects hindering bacterial growth. As a former Invited Forum Editor and Author of *Antioxidant & Redox Signaling*, I clearly understand and adhere to the journal's policy of scientific excellence. In this vein, I am confident that the work by Wareham *et al*. fits those standards while making a remarkable scientific contribution. The few questions/concerns raised by the reviewers are not of enough scientific substance to reject sound work based on a substantial and comprehensive amount of evidence. The key issue from this work is that perturbations of the proton motive force elicited by CORM-401 are matched by enhanced respiration leading to membrane polarization, which in turn drives the uptake of CORM-401 by bacteria, poisoning them by its intracellular accumulation. This seminal effect facilitates the intracellular action of CORM-401 on multiple targets leading to growth arrest. Therefore, in the interests of science, I take full responsibility to rescue this work from rejection.

**Giancarlo Biagini** (*giancarlo.biagini@lstmed.ac.uk*): I am a qualified reviewer (per *Antioxid Redox Signal* 16: 293–296) and move to rescue this article that was rejected during the regular peer review process after reviewing all versions of the article and detailed reviewer comments. The article investigates the mechanism of action of a CO-releasing molecule and describes biochemical, cellular bioenergetic, and transcriptional responses of *E. coli* to CORM-401. The innovation of the article is that these data are the first to describe the pharmacodynamics of this second-generation CORM. The findings suggest a pleiotropic mechanism of action, which includes disruption of respiratory chain components and of membrane integrity in terms of ion transport/homeostasis. It is likely that the disruption of these two biological functions is linked. One of the referees questions the demonstration that CORM-401 stimulates respiration. However, this is clearly demonstrated in Figure 7. The experiments show the use of the open O_2_ electrode, widely used and often in preference to the closed system, as one that has the ability to set the dissolved O_2_ tension of the chamber, which clearly demonstrates stimulation of O_2_ consumption following the introduction of CORM-401. The positive control experiment using KCN clearly indicates that the system is working normally. *In vivo* data are required to establish the development potential of molecules, but in this case, this investigation is first attempting to determine the molecular mechanisms underpinning the activity of this class of compounds. It is therefore not normal practice to conflate the two issues. Therefore, in the interests of science, I take full responsibility to rescue this work from rejection.

**James Imlay** (*jimlay@illinois.edu*): I am a qualified reviewer (per *Antioxid Redox Signal* 16: 293–296) and move to rescue this article that was rejected during the regular peer review process after reviewing all versions of the article and detailed reviewer comments. I have closely read the article and the reviewers' comments. My overall reaction is that the article is sound, and I advise that a decision be made to accept the article. I was surprised by the outcome. I would have predicted that toxicity was mediated by the usual action of CO. The loss of viability in Figure 2 was enough to alert me that I was wrong. This is nice work. A reviewer requested *in vivo analysis*, but because these compounds impact host cells, a study of CORM-401 impact in host animals would be large and complex. Another wrote: *authors should determine O_2_ consumption rate.* This method is not new. Standard measurements of consumption (Fig. 7B) are problematic when rates are low. However, in Figure 7C and 7D, the reader can immediately see that CORM-401 and carbonylcyanide *m*-chlorophenylhydrazone cause an increase in oxygen consumption, as evidenced by the lower steady-state oxygen level. The same reviewer wrote: *oxygen consumption or even OCR could increase due to several processes other than respiration.* I disagree with the reviewer's suggestion that nonrespiratory processes could accelerate oxygen consumption. *E. coli* lacks nonrespiratory enzymes that employ molecular oxygen as a substrate as this facultative bacterium must manage biosynthesis without oxygenases. Indeed, if one knocks out respiratory oxidases, oxygen consumption essentially ceases. Therefore, in the interests of science, I take full responsibility to rescue this work from rejection.

**Nigel Robinson** (*nigel.robinson@durham.ac.uk*): I am a qualified reviewer (per *Antioxid Redox Signal* 16: 293–296) and move to rescue this article that was rejected during the regular peer review process after reviewing all versions of the article and detailed reviewer comments. Carbon monoxide-releasing molecules (CORMs) might be a new class of antimicrobials, with the looming threat of antimicrobial resistance making work to explore such options pertinent. In this study, multiple approaches have explored facets of bacterial physiology (respiration, transport, and membrane integrity) and molecular cell biology (including extensive expression profiling) to carefully tease apart the mechanism(s) of bacterial growth inhibition by CORM-401. The resulting observations do not mirror the effects of related ruthenium-containing CORMs, nor does exposure of cells to CO alone mimic the effects of CORM-401: these data thus point to features of the compound other than solely the liberated CO exerting inhibitory effects, with manganese, as opposed to ruthenium, appearing to be one crucial distinction. The work is a *tour de force*; in short, the action of CORM-401 is multifaceted and includes not only CO binding to respiratory oxidases but also manganese hyperaccumulation with effects on the homeostasis of other metals (such as zinc), ionic imbalance and loss of membrane integrity, and defined regulons. The extent of functional studies was questioned, yet the work is rich in a diversity of types of functional studies; these assays have demanded a high degree of technical skill in using a wide range of methodologies. Evidence that the compound stimulates respiration was questioned. However, bacterial respiration rates have been determined from measurements of oxygen concentrations and oxygen diffusion rates. Editorial comments note the lack of *in vivo* studies: it may be felt that ultimately the compound should be used in animal models of infection, but the modest effectiveness of this CORM as an antimicrobial agent suggests that animal studies are not justified (or perhaps ethical). Therefore, in the interests of science, I take full responsibility to rescue this work from rejection.

In this study, we present the most detailed microbiological analysis to date of a non-Ru CORM and contrast its effects with the potent actions of CORMs-2 and −3, whose actions are probably explicable, in part, by the biological chemistry of the accumulated Ru. Although less potent as an antibacterial agent, CORM-401 is extensively accumulated by bacteria, increases oxygen consumption and membrane polarization in an uncoupler-like manner, and triggers myriad effects, including osmotic and envelope stresses.

## Results

### CORM-401 releases CO under bacterial growth conditions

**FIG. 1.**
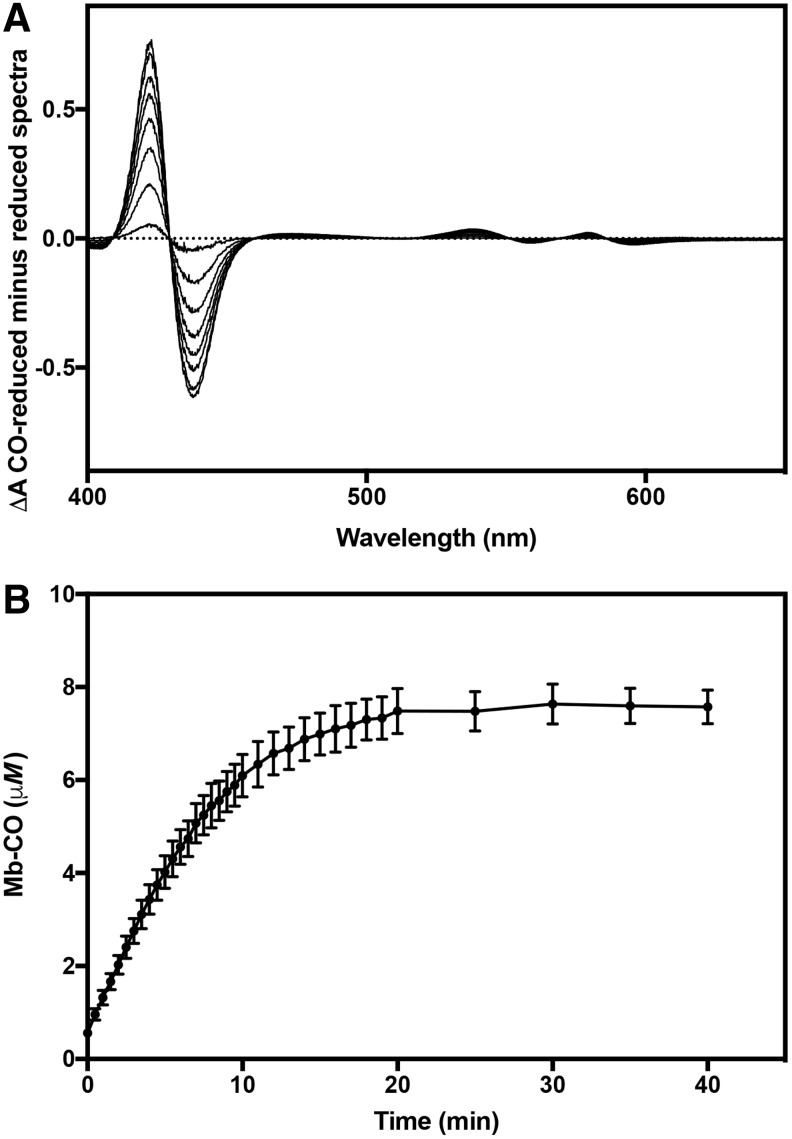
**CO release from CORM-401 in bacterial growth medium. Assays were performed in Evans minimal medium (with glucose as sole carbon source) in the presence of excess dithionite at 37°C. (A)** CO difference spectra showing the conversion of myoglobin (15 μ*M*) to the carbonmonoxy-ferrous species in the presence of 3 μ*M* CORM-401. **(B)** Time course of CO release from CORM-401 in medium at 37°C. Under these conditions, CORM-401 releases 2.5 mole equivalent of CO with a t_1/2_ of 4.5 min. Data are means of three biological repeats ± SEMs. CORM, carbon monoxide-releasing molecule.

Medium or buffer composition and the presence of oxidants, reductants, and a CO acceptor (such as a heme protein) are important determinants of the rates and extent of CO loss from CORMs (16, 39). We therefore characterized CO release from CORM-401 *in vitro* under our conditions using ferrous myoglobin assays in which maximum sensitivity was achieved by monitoring the Soret bands in CO difference spectra (39) (Fig. 1A). In defined (Evans) growth medium, at physiological temperature (37°C) and pH (7.4), 2.5 mol equivalent of CO was released to myoglobin with a half-time of 4.5 min. In 0.1 *M* KPi buffer at 37°C, the half-time was 5 min and the yield of CO was 2.4 mol/mol; at 20°C, the rate was slower (Supplementary Fig. S1; Supplementary Data are available online at www.liebertpub.com/ars). Fayad-Kobeissi *et al.* (16) reported that the yield of CO increased as the myoglobin:CORM-401 ratio increased, with 2.6 mol observed at a ratio of 5:1. This is in close agreement with our values (Fig. 1B and Supplementary Fig. S1).

Although sodium dithionite facilitates CO release from CORM-3, CORM-401 is able to release CO spontaneously in the absence of dithionite with oxyhemoglobin as a CO acceptor (39). The CO release kinetics in Figure 1 demonstrate the rapid release from CORM-401 of CO in growth medium; whether CO is released before, during, or after CORM uptake (see Fig. 3), the gas will be available in the following bacterial experiments.

### CORM-401 toxicity is dependent on the carbon source for growth

**FIG. 2.**
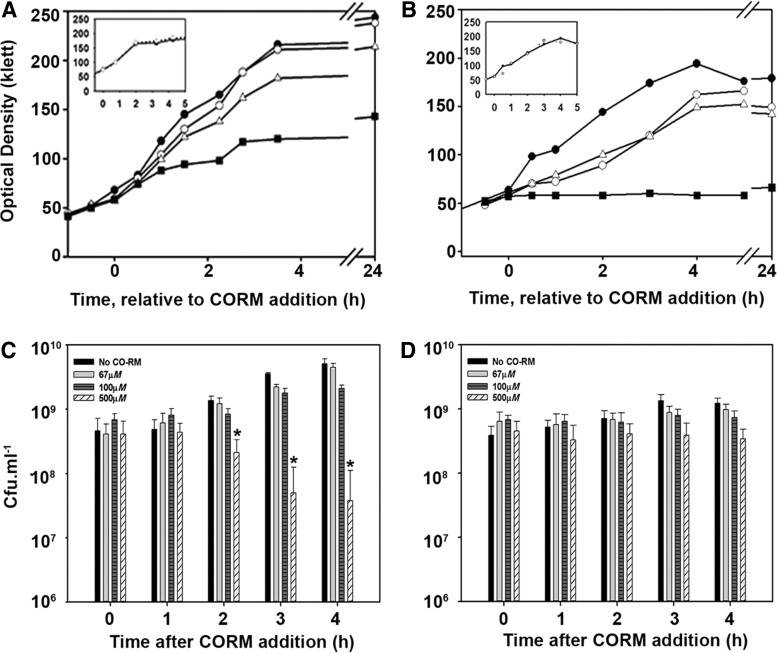
**CORM-401 inhibits growth of** ***Escherichia coli*** **and is bactericidal to glucose-grown cells.** Cells were grown to the exponential phase before addition of CORMs at time = 0. **(A)** Growth supplemented with glucose in the absence of CORM-401 (*closed circles*) and after addition of 10 μ*M* (*open circles*), 67 μ*M* (*open triangles*), and 500 μ*M* (*squares*) CORM-401. **(B)** Growth supplemented with succinate, symbols as above. *Insets* in **(A)** and **(B)** Growth with 500 μ*M* DTC/MnSO_4_ on glucose and succinate showed no deleterious effect on growth of cells. **(C)** Viable counts at hourly intervals of cells growing on glucose. **(D)** Viable counts of cells growing on succinate. Following the addition of CORM-401 to *E. coli* MG1655 at OD_600_ 0.6, cells were incubated for 90 min before sampling; * indicates *p* ≤ 0.008. Data are representative of three biological repeats.

The effect on bacterial growth was determined by adding CORM-401 to mid-exponential phase cultures in replicate experiments (representative cultures are shown in Fig. 2A, B). CORM-401 slightly perturbed growth of cells on glucose at 67 μ*M* and significantly slowed growth only at 500 μ*M* (Fig. 2A). When CO is liberated from CORM-401, an Mn(II) salt and a dithiocarbamate (DTC) ligand remain. In minus-CO control experiments, a combination of Mn(II) sulfate and sarcosine DTC was added to final concentrations of 500 μ*M* with no deleterious effect on growth (Fig. 2A, inset).

We hypothesized that succinate-grown cells would be perturbed by CORM-401 to a greater extent than on glucose due to physiological reliance on heme terminal oxidases that are inhibited by CO. Indeed, growth on succinate was inhibited to a greater extent by CORM-401 (Fig. 2B); unlike glucose-grown cells, cells failed to grow on succinate with 500 μ*M* CORM-401 (Fig. 2B). Although inhibition of oxidase function may contribute to this distinction, this article reveals a multitude of effects on unrelated cellular functions. The control compounds again showed no deleterious effect on growth (Fig. 2B, inset). To dissect the effect of CO gas and the CORM, we tested the addition of a CO-saturated solution on growth of cell cultures. Due to limitations of CO solubility and the undesirability of adding large volumes of gas solutions to cultures, a final concentration of 100 μ*M* was used; no inhibition of growth was observed (data not shown), as shown before (69). Thus, the observed effects of CORM-401 on growth are, in part, attributed to the metal co-ligand fragment of the compound and not the CO alone as previously supposed.

Bactericidal activity of the compound was assessed by viable counts. Note, however, there is no useful correlation between biomass as assessed by optical density (light scattering) measurements (Fig. 2A, B) and viable cell numbers as assessed by colony counting after dilutions (Fig. 2C, D). Light scattering certainly does not give information on the number of cells (17, 32). Although glucose-grown cultures increased in total biomass at <100 μ*M* CORM-401, viability on agar decreased at 500 μ*M* after 2 h (Fig. 2C). In contrast, cells grown on succinate (Fig. 2D) retained their viability on agar, even at 500 μ*M* CORM-401, despite a lack of increase in biomass (Fig. 2B). The apparent discrepancy in these two media might result, for example, from the clumping of cells in glucose (leading to an artifactually low cell count) or the ability of succinate-grown cells to retain viability after removal from the CORM, although being inhibited within the CORM-supplemented culture.

### CORM-401 accumulates to millimolar concentrations in the bacterial cytoplasm and binds DNA

We hypothesized that the loss of viability of glucose-grown cells (Fig. 2C) might be due to substrate-dependent differences in CORM uptake. CORMs-2 and −3 are accumulated by bacteria, as inferred from intracellular Ru concentrations (9, 27, 38). Therefore, intracellular accumulation of CORM-401 was measured using inductively coupled plasma mass spectroscopy (ICP-MS) (38). However, the metal in CORM-401 is Mn, itself a component of all cells, and so we sought elevated accumulation of this metal relative to no-CORM controls. Cells growing on glucose or succinate in the presence of CORM-401 at two concentrations were assayed over time and concentrations of manganese were determined in cell pellets, using literature values for cell size and volume to estimate cellular concentrations.

**FIG. 3.**
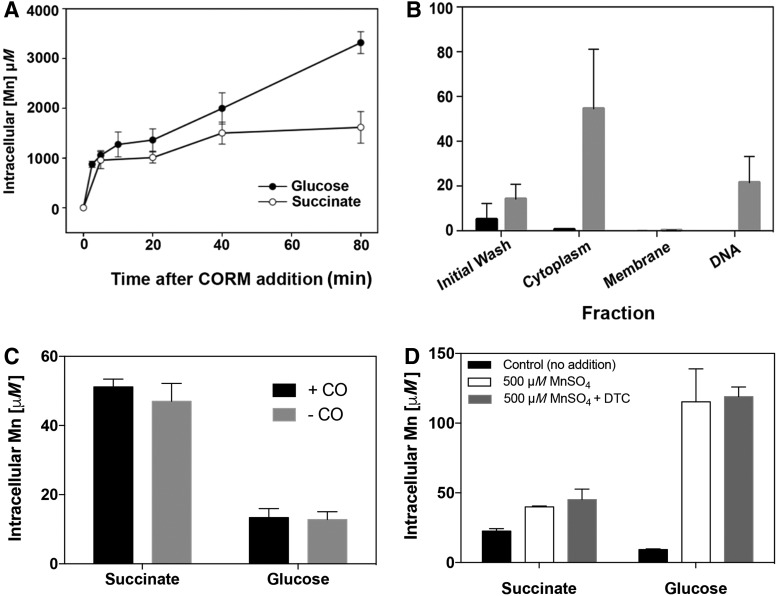
**Manganese levels in cells treated with CORM-401 or control compounds. (A)** Intracellular Mn levels in *E. coli* grown on 20 m*M* glucose (*closed circles*) or 20 m*M* succinate (*open circles*), then treated with CORM-401 (final concentration 67 μ*M*); *n* = 3, ± SEM. **(B)** Localization of Mn following addition of 67 μ*M* CORM-401 (*gray bars*) to *E. coli* MG1655 subcellular fractions. Black bars show Mn amounts in each fraction with no additional CORM-401. **(C)** Mn levels in cells incubated for 80 min in the presence (*black bars*) or absence (*gray bars*) of a saturated CO solution (∼500 μ*M*, final concentration). **(D)** Mn levels in cells before additions (*black bars*) or after adding 500 μ*M* MnSO_4_ ±67 m*M* DTC. *n* = 3, ± SEM. Note the different scales in **(A–D)**.

When cells were grown on glucose with 67 μ*M* CORM-401, manganese accumulated biphasically (Fig. 3A, closed circles), rapidly within 2.5 min, then more slowly over 80 min to reach an intracellular concentration of ∼3.5 m*M* manganese. In contrast, uptake by succinate-grown cells was monophasic, reaching ∼1.5 m*M* after 80 min (Fig. 3A, open circles). Thus, in glucose media, bacteria accumulate more CORM-401 (Fig. 3A). Since 500 μ*M* CORM-401 was needed to significantly slow growth, we also tested this concentration and assayed cell pellets after 80 min of growth; in cells grown on glucose or succinate, the values found in cell pellets were 16.5 ± 0.69 m*M* and 10.9 ± 0.55 m*M*, respectively. The data sets are statistically different (*p* < 0.05). Thus, irrespective of the CORM concentration in media, bacteria accumulate more CORM-401 in glucose media and suffer reduced viability (Fig. 2C). Note, however, most of the accumulated manganese will be bound and buffered such that the exchangeable concentration within living cells will be much lower than the maximum of 3.5 m*M* that we recorded. Therefore, an apparent concentration of, say, 3.5 m*M* manganese is better expressed as 0.7 × 10^7^ atoms/cell (see the Materials and Methods section).

To determine the fate of accumulated manganese, cultures exposed to 67 μ*M* CORM-401 were harvested and fractionated to give soluble, membrane, and genomic DNA fractions, which were retained and quantitatively analyzed for manganese by ICP-MS. As shown in Figure 3B, manganese was found predominantly not only in the cytoplasm but also in DNA. CORM-401 itself is unlikely to coordinate DNA, but pyridine reacts to give Mn(CO)_3_(py)(DTC), as observed by IR spectroscopy (7), suggesting that an N base in DNA could behave similarly. A protein histidine residue is a candidate for binding of CORM-3 (59).

It was important to define the active species of CORM-401; this might be CO released from the compound, the residual Mn(II) salt, or the DTC ligand. In the absence of CORM, the Mn levels of succinate-grown cells were higher than for glucose-grown cells (Fig. 3C) (note the different ordinate scales in Fig. 3A, C, D). However, metal levels were not influenced by CO in solution, even at 500 μ*M*, after 80 min of incubation. Adding MnSO_4_ (500 μ*M*) increased the intracellular Mn levels, as expected, but a combination of MnSO_4_ and DTC was without further effect. We conclude that the modest increases observed in the control experiments in Figure 3C and D demonstrate that CORM-401, but not the control species (Mn, CO, DTC), significantly elevates intracellular Mn pools.

### Exposure to CORM-401 leads to transient changes in transcription of multiple gene groups

**FIG. 4.**
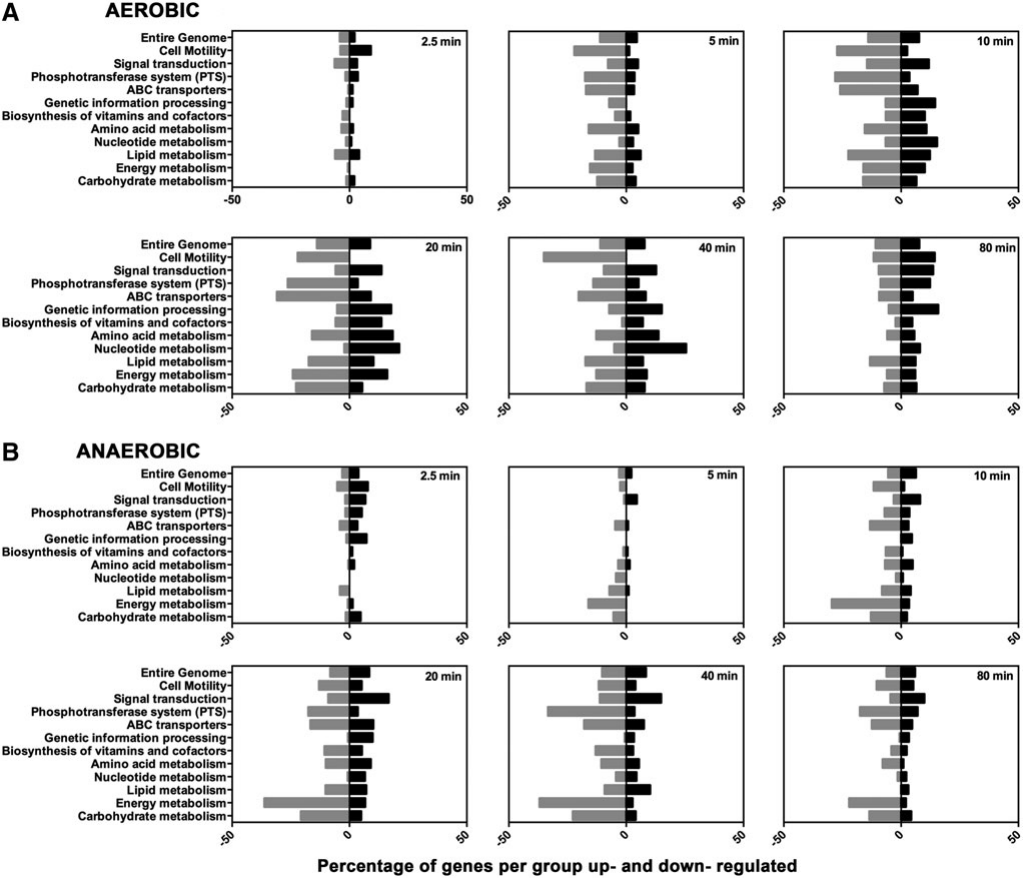
**Functional categories of genes affected by CORM-401 under aerobic and anaerobic conditions.** Genes are grouped according to functional categories. The bars show the percentage of genes in each group that exhibit significantly altered gene expression (*i.e*., where fold changes are ≥2 and ≤0.5) under **(A)** aerobic conditions and **(B)** anaerobic conditions. Gene changes are shown as genes upregulated (*right*, *black bars*) and downregulated (*left*, *gray bars*) in each group.

The accumulation of CORM-401 to strikingly high levels and correlation with bacterial killing (Figs. 2, 3) prompted further analysis of global responses to this CORM. We therefore conducted transcriptomic analyses on CORM 401-treated cells in glucose medium. The concentration of CORM selected (67 μ*M*) led to minor growth perturbation. Lethal concentrations of CORM were avoided: we sought transcriptomic responses to stress responses, rather than cell death. CORM-401 altered expression of numerous genes in distinct classes (Fig. 4). Following standard practice, we retained, for our initial analysis, genes exhibiting a fold change ≥2-fold up or ≥2-fold down (the latter representing a change of ≥0.5 of the control transcript level). We then performed in-depth analyses of the transcriptomic response using the statistical modeling approach of TFInfer.

The response was transient both aerobically and anaerobically. Aerobically, 23% of the genome was significantly up- or downregulated within 40 min (Fig. 4A). Anaerobically, although the magnitude of gene changes across many of the categories was smaller, and the response slightly delayed, the pattern in the response was again transient; at 40 min, gene changes were highest with 18% of the entire genome changing (Fig. 4B).

Aerobically, the most marked changes were in genes involved in motility and energy, carbohydrate, and nucleotide metabolism (Fig. 4A). The most altered category was motility; after 40 min of exposure to CORM-401 in aerobic conditions, 35% of genes in this category were downregulated, consistent with the compromised motility of cells treated with a solution of CO (38). However, we found that motility (swarming) of cells was not diminished by CORM-401 (see the CORM-401 causes induction of the Cpx and Bae regulons, altering the expression of Spy and CpxP proteins but without measurable membrane damage to cells section). Energy metabolism genes were transiently affected by the addition of CORM-401 under aerobic conditions; genes were downregulated progressively over time and reached a minimum at 20 min where 24% of energy metabolism-related genes were downregulated (Fig. 4A). Anaerobically, the gene categories most affected were those involved in energy metabolism, carbohydrate metabolism, phosphotransferase system genes, and motility (Fig. 4B). Genes involved in energy metabolism were more highly downregulated anaerobically than aerobically (Fig. 4B and Fig. 6). Signal transduction genes and genetic information processing genes were highly upregulated: 17% of signal transduction genes were upregulated after 20 min.

Genes are described subsequently according to their functional characteristics. However, Supplementary Table S1 presents a list of the thirty most highly upregulated genes, both aerobically and anaerobically.

#### Modeling of transcriptomic data

**FIG. 5.**
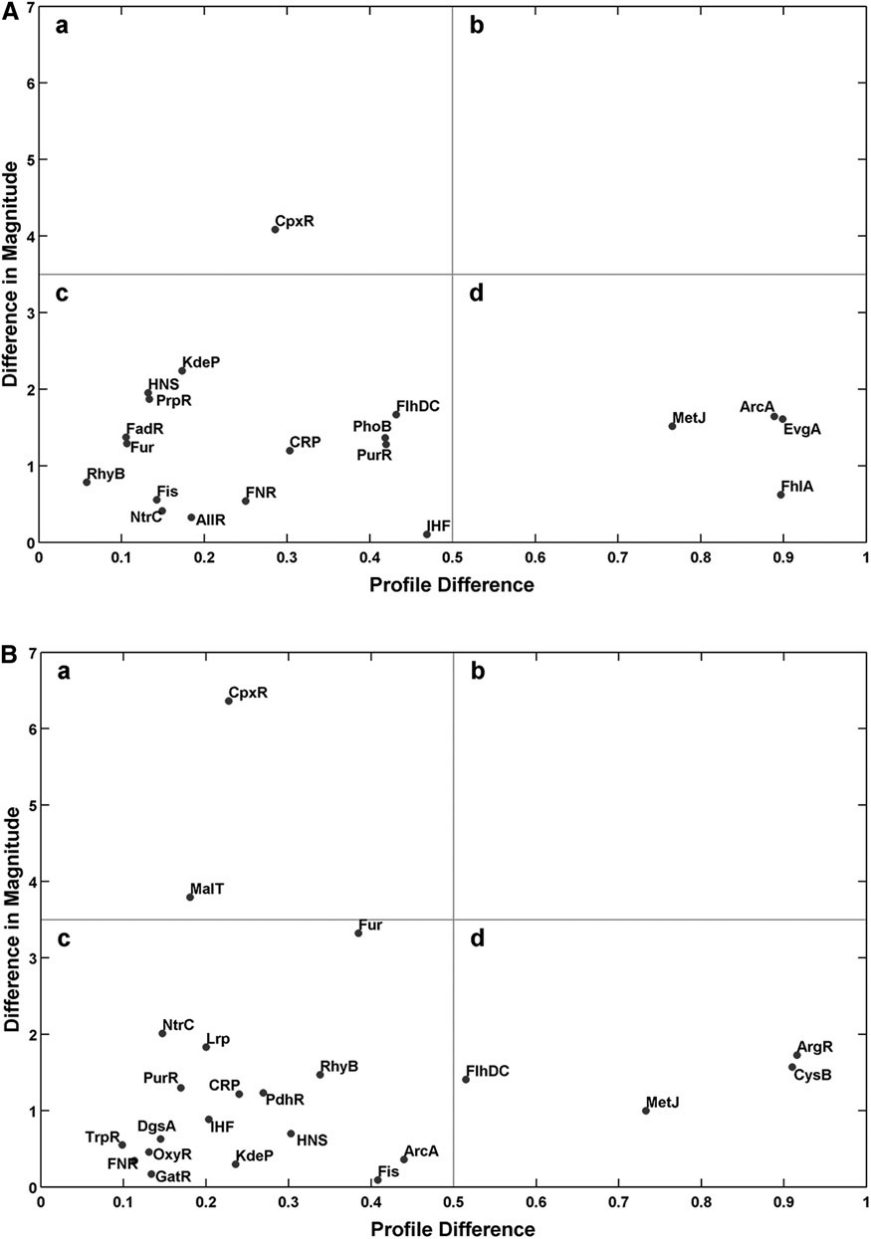
**TFInfer correlation profiles (coherence plots) showing TFs involved in the response to CORM-401** ***versus*** **CO gas in** ***E. coli*** **cells.** The *x*-coordinate of each point represents the profile difference between CORM-401 and CO treatments, computed as 1 minus the absolute Pearson correlation coefficient between the two profiles); the *y*-coordinate represents the change in magnitude of the response (computed as the difference of the norm of the two profiles). Data from (**A**, *top*) aerobic and (**B**, *bottom*) anaerobic conditions are shown. TFs whose response is similar with both CORM-401 and CO gas, both in magnitude and kinetics, will be located near the origin of each coherence plot in quadrant c, while TFs in quadrant b of each plot respond differently in both kinetics and amplitude. For example, CpxR, both aerobically and anaerobically, has a similar response in terms of the shape of the profile, but different magnitudes in **(A)** and **(B)**, while ArcA **(A)** and ArgR **(B)** show similar magnitudes, but major differences in response profiles. TF, transcription factor.

To analyze the underlying transcription factors (TFs) responsible for the transcriptome data, we used TFInfer (2, 57, 69), a Bayesian statistical method that integrates gene expression data with regulon information (culled from Regulon DB or EcoCyc) to identify TF activity profiles that aid in understanding of the raw transcriptional changes. We ran TFInfer separately on the CORM-401 (this work) and CO gas data sets (69) to identify differences in the magnitude and kinetics of the response to the two stimuli. Figure 5 summarizes the differences between two sets of TFInfer data using coherence plots [introduced in (69)]; the abscissa (*x*-coordinate) of each point (labeled with the TF identity) represents the profile difference between the two conditions, computed as 1 minus the absolute Pearson correlation coefficient between the two profiles, while the ordinate (*y*-coordinate) represents the change in magnitude of the response (computed as the absolute difference of the norms of the two profiles). Hence, TFs whose response is similar both in magnitude and kinetics are located near the origin of the coherence plot, while TFs in the top right corner of the plot respond differently in both kinetics and amplitude.

In this analysis, we identified several key regulators whose activities underlie the effects described later. Thus, CpxR appears in quadrants “a” of Figure 5 (A, aerobic and B, anaerobic), hence exhibiting correlated temporal profiles, but a large difference in response magnitude. CpxR is a member of the two-component regulatory system CpxA/CpxR that combats extracytoplasmic protein-mediated toxicity by increasing the syntheses of the periplasmic protease DegP and CpxP protein. However, the response regulator Fis, which is involved in maintenance of nucleoid structure and other functions, including biofilm formation, motility, and chemotaxis, is in both quadrants “c” of Figure 5, indicating that its response is similar in magnitude and kinetics when cells are exposed to CO gas or CORM-401 in aerobic or anaerobic conditions. As a further example of the correlation analysis, the coherence plot also reveals that FhlA (the transcriptional activator of the formate hydrogenlyase system, quadrant “d” of Fig. 5, top, aerobic) responds similarly in terms of the activity profile when cells are exposed to CORM-401 or CO gas, but the magnitudes of the responses are dissimilar and FhlA does not appear in the anaerobic analysis. (Note that FlhA, required for flagellar biosynthesis, also appears in these analyses as a pertinent TF, but its activity is less altered in the aerobic and anaerobic states.) Each point on the plot has both horizontal and vertical error bars associated with it, which take into account the uncertainty in the inferred TF activities derived from TFInfer. In Figure 5, these error bars have been omitted to reduce visual clutter; error bars are shown in Supplementary Figure S2.

#### CORM-401 perturbs respiratory gene expression

Energy metabolism was significantly altered, both aerobically and anaerobically, in response to CORM-401 (Fig. 4). The TFInfer coherence plot reveals two TFs that regulate genes in central respiratory metabolism: Fnr (fumarate nitrate reduction regulator) and ArcA. While Fnr was less perturbed (being in quadrant “c” of Fig. 5A, B), ArcA responded differently under each condition (lying in quadrant “d” of Fig. 5A, but in quadrant “c” of Fig. 5B). ArcAB, a two-component system, indirectly senses oxygen, in part, *via* the redox state of the quinone pool (1). Under anoxic or microaerobic conditions, ArcB autophosphorylates, then transphosphorylates ArcA through a phosphorelay, increasing the affinity of ArcA for its DNA targets (26). Phosphorylated active ArcA (ArcA-P) then represses expression of genes involved in aerobic respiration (*e.g*., electron transport enzymes, cytochrome *bo’*, and the Krebs cycle enzymes) and activates genes involved in fermentative metabolism and cytochrome *bd.* Thus, in a mutant lacking oxidase function, as in the absence of oxygen as electron acceptor, the aerobic expression of ArcA-P-activated genes such as *cydAB* is elevated, but that of ArcA-P-repressible genes such as *cyoABCDE* (encoding the heme-copper oxidase) is lowered (25) because the quinone pool is trapped in a reduced form and unable to inhibit the autokinase activity of ArcB.

**FIG. 6.**
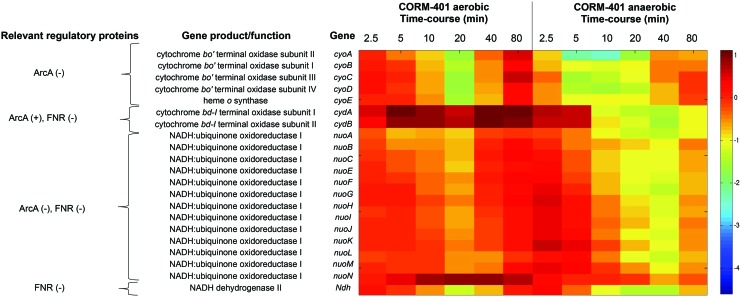
**Differential expression of genes involved in the respiratory chains both aerobically and anaerobically in response to 67 μ*M*** **CORM-401.** The heat map quantifies the changes elicited in selected genes; the heat scale at the *right* is expressed as the natural logarithm of the fold change. To see this illustration in color, the reader is referred to the web version of this article at www.liebertpub.com/ars

Indeed, Figure 6 shows that CORM-401 mimicked microaerobic/anaerobic conditions even in the presence of oxygen; it very strongly increased expression of *cydAB* (encoding cytochrome *bd-I*) presumably reflecting respiratory inhibition. Davidge *et al.* also reported an increase in *cydAB* transcripts in response to CORM-3 (9). However, anaerobically, the *cydAB* genes were downregulated after 5 min. In contrast, genes encoding the cytochrome *bo'*-type oxidase (*cyoABCDE*) were, under aerobic conditions, transiently downregulated by approximately fivefold after 5 min. This is consistent with repression of *cyo* expression by phosphorylated (active) ArcA. Anaerobically, the *cyo* operon was consistently downregulated by CORM-401; *cyoA* was 10-fold downregulated after 10 min. Fold changes relating to respiratory changes are given in Supplementary Figure S3A.

Figure 6 also shows other respiratory genes. The *nuo* genes encode a multisubunit, proton-translocating NADH dehydrogenase; all were upregulated by CORM-401 under aerobic conditions together with the oxidase genes. The initial CORM-401-induced increases in *cyo* expression (0–5 min) and the sustained increase in *nuo* expression (0–80 min) under aerobic conditions are not consistent with the anticipated repression of these systems by Arc alone; indeed, *nuo* expression is regulated in a complex manner not only by ArcA but also by NarL, Fnr, IHF, and other factors, including C_4_ dicarboxylates (5). In contrast to the effects of CO gas (69), expression of *ndh*, encoding NADH dehydrogenase II, was upregulated both aerobically and anaerobically for the first 5–10 min after CORM-401 exposure (Fig. 6).

#### CO released from CORM-401 is targeted to terminal oxidases *in vivo*

**FIG. 7.**
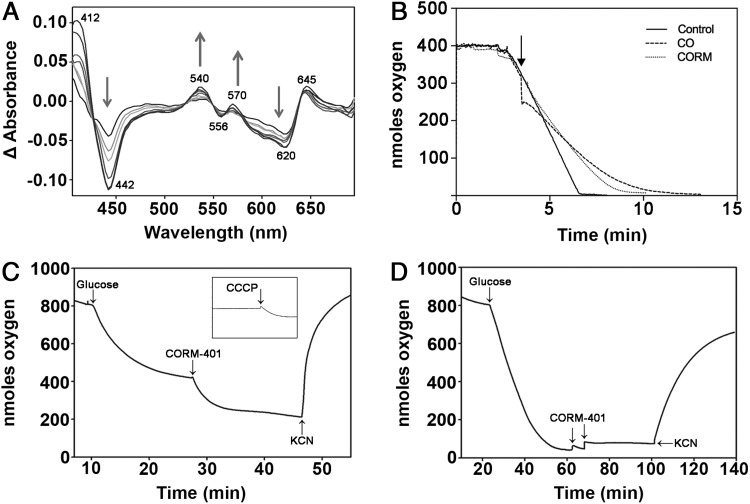
**CO released from CORM-401 binds terminal oxidases in whole cells, but exhibits uncoupler-like activity on respiration**. **(A)** Cells were grown in LB media to the exponential phase and concentrated in PBS to an OD of ∼55. CORM-401 was added to a final concentration of 100 μ*M*. CO difference spectra were recorded over 15 min; *arrows* show the direction of absorbance increase or decrease in successive scans. **(B)** Membrane particles (60 μg protein/mL) from wild-type *E. coli* were added to a closed electrode chamber. Respiration was stimulated by addition of NADH and when air saturation had reached ∼75% of the initial (*arrow*), CO saturated solution (*dashed line*) or CORM-401 (final concentration 100 μ*M*) was added (*gray dotted line*). Respiration of membranes in the absence of any compounds was followed as a control (*solid line*). **(C)** Cells were grown to the mid-exponential phase (OD_600nm_ ∼0.6) in Evans medium and resuspended in Tris-HCl buffer, pH 7.4, before analysis in an open oxygen electrode. Where indicated by *arrows*, glucose was added to stimulate respiration and cells respired until a steady state was reached before addition of 100 μ*M* CORM-401. *Inset* shows 10 μ*M* CCCP added under equivalent conditions. **(D)** Partial inhibition of respiration was observed at low oxygen tensions when CORM-401 was added twice (*arrows* marked CORM-401). In **(C)** and **(D)**, KCN was added at the *arrows* at the end of the experiment to the chamber to a final concentration of 1 m*M* to fully inhibit respiration. All data are representative of three biological repeats.

Changes in respiratory gene expression suggested interference with respiration, so we assessed CO targeting of the oxidases using spectrophotometry on intact cell suspensions. CO bound rapidly to terminal oxidases upon addition of 100 μ*M* CORM-401 (Fig. 7A). In the first scan, the 645 nm signal corresponds to the absorbance maximum of CO-ligated cytochrome *d*, and the 620 nm trough indicates removal of ferrous cytochrome *d* from the difference spectrum (9, 27). Subsequently, a peak at 412 nm appeared, suggesting slower binding of CO to cytochrome *o.* The developing trough at 442 nm has contributions from the ferrous hemes of cytochromes *o*, *d*, and *b*_595_ (27, 56). More slowly formed were the α/β bands at 540 nm, 556 nm, and 570 nm with contributions from multiple CO-reactive hemes. Thus, CO is released almost immediately upon addition to cells and is initially targeted to the cytochrome *bd*-type oxidase, coincident with upregulation of *cydAB* transcripts in aerobic cells (Fig. 6 and, Supplementary Fig. 3A).

#### CORM-401 stimulates respiration

In view of the expected (29) and observed targeting of respiratory oxidases by CO released from CORM-401 (Fig. 6), we measured directly the effects of CORM-401 on respiration. Bacterial membranes were incubated in a chamber closed with a lid preventing inward diffusion of oxygen so that oxygen consumption decreases dissolved oxygen. Respiration was stimulated with 6.25 m*M* NADH and followed until ∼75% air saturation (shown by the arrow in Fig. 7B), where 100 μ*M* CORM-401 or CO-saturated solution was added. Under these conditions, CORM-401 inhibited membrane respiration by up to 32% and equimolar CO gas inhibited respiration by up to 48%. However, the periods of observation were limited by oxygen depletion from the chamber.

To allow longer observations on intact bacteria, in which the duration of the measurement is not limited by the amount of O_2_ initially present in the liquid, we used the well-established open system, allowing inward O_2_ diffusion to balance oxygen consumption. In such a chamber (Fig. 7C) (10), the respiration rate at steady state is proportional to the difference between the equilibrium concentration of O_2_ and the steady-state concentration of O_2_ in solution; in contrast, in a closed system (Fig. 7B), the respiration rate is proportional to the negative slope of the plot of O_2_ concentration *versus* time. To stimulate respiration, glucose was added (Fig. 7C), whereupon the equilibrium concentration of O_2_ fell. Respiration was followed at a constant stirring speed (and thus oxygen transfer rate) adjusted to give a near-steady state around 50% air saturation. When 100 μ*M* CORM-401 was added to the chamber (Fig. 7C), respiration clearly increased as evident in the further fall in dissolved O_2_ concentration. This pattern resembles increases in respiration rates induced by substrate provision in *Klebsiella* (10). A similar increase in respiration rate was observed (28) when this concentration of CORM-401 was added to endothelial cells. The theory for O_2_ transfer into a suspension culture is well known [for references, see (10)]: provided that the liquid–gas interface surface area is constant, the rate of O_2_ transport from the gas to the liquid is given by *v = K_L_a(T_G_-T_L_)*, where *v* is the rate of respiration, *K* is a constant that depends on the experimental volume, surface area, and temperature, *T_G_* is the molar concentration of O_2_ in the liquid when equilibrated with air, and *T_L_* is the O_2_ concentration in the liquid. In the nonproliferating cell suspensions used here, the slow decline in dissolved O_2_ after a near-steady state was reached was neglected. We measured a *K_L_a* (11) of 0.23 min^−1^ (SD 0.06, 10 measurements) and a glucose-stimulated respiration rate of 215 nmol O_2_.min^−1^.mg^−1^ (SD 78, 13 measurements).

In the experiment shown in Figure 7C, respiration increased by 50 % after adding 100 μ*M* CORM-401, typical of numerous experiments. This increase is similar to that observed before (28, 52). As a control, 1 m*M* KCN (a potent respiratory inhibitor) was added to the chamber (Fig. 7C) when steady state had been reached after adding CORM; dissolved oxygen then increased abruptly to prerespiration levels, indicating respiratory inhibition as expected. This pattern resembles decreases in the respiration rate induced by substrate exhaustion in *Klebsiella* (10). The CORM 401-stimulated oxygen consumption can be assigned to cellular respiration since it is cyanide sensitive. The stimulation by CORM-401 was further investigated by using the same cell suspension (as Fig. 7C), but 10 μ*M* carbonyl cyanide *m*-chlorophenyl hydrazone (CCCP), a classical uncoupler of respiration, was added to the chamber (Fig. 7C, inset). CCCP mimicked the effects of CORM-401; thus, both compounds stimulate oxygen consumption rates of *E. coli* cells.

In these experiments, dissolved oxygen was >50% saturation (∼100 μ*M*) at the point of adding CORM. However, maximal inhibition of respiration typically occurs at high CO:O_2_ ratios since CO is a competitive inhibitor of terminal oxidases with oxygen; CO:O_2_ ratios of 4:1 to 20:1 are used in photochemical action spectra (6). Thus, to maximize the potential inhibition of respiration by CORM-401, the stirring rotor speed was decreased to achieve a lower (but not zero) oxygen concentration in the chamber after stimulating respiration with glucose (Fig. 7D). On adding CORM-401, a very small transient inhibition was seen. A second addition of 100 μ*M* CORM-401 (arrow 2) produced mild, but prolonged, inhibition. To confirm these findings, 1 m*M* KCN was again added to the chamber and substantial inhibition followed. In the experiments of (28), 300 μ*M* CORM-401 was required to inhibit respiration by endothelial cells. In summary, CORM-401 is not an effective inhibitor of respiration, even at low oxygen tensions, but stimulates respiration; such stimulation was described as uncoupling in endothelial cells (28).

#### CORM-401, like CCCP, polarizes the membrane in whole cells

**FIG. 8.**
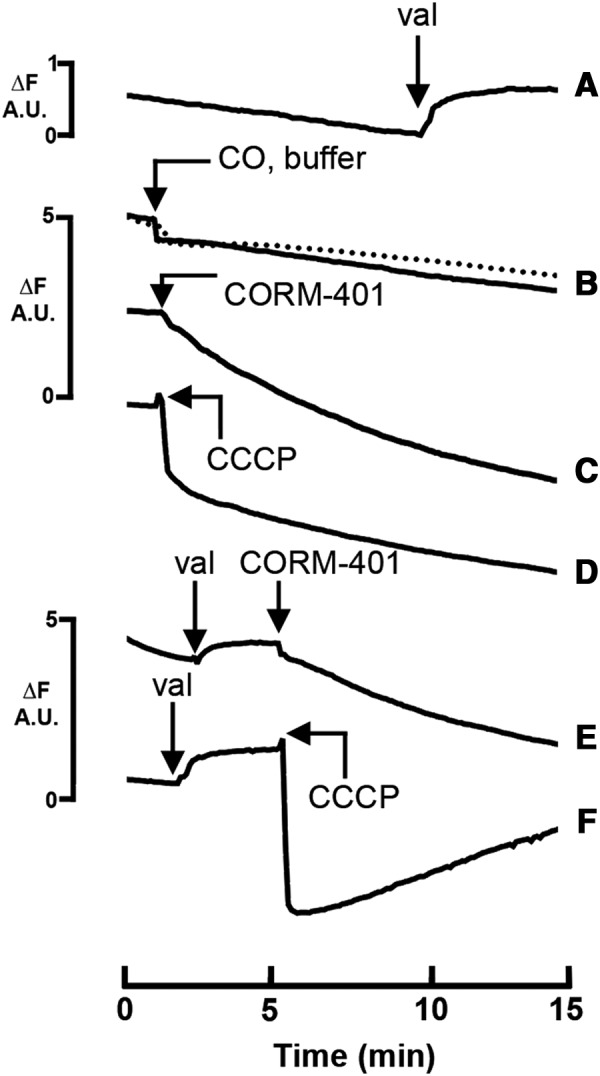
**CORM-401 and the classical uncoupler CCCP cause polarization of the membrane in whole cells.** *E. coli* cells were grown to the exponential phase and resuspended in 5 m*M* HEPES buffer to a final OD_600_ of 0.6. Cells were incubated with 0.1 *M* KCl and 10 m*M* glucose before incubation with 0.4 μ*M* DiSC3(5). Additions were as follows: **(A)** 1 μ*M* valinomycin (val); **(B**) 50 μ*M* CO (*solid line*) or buffer (*dotted line*); **(C)** 50 μ*M* CORM-401; and **(D)** 1 μ*M* CCCP. Trace **(E)** shows additions of 1 μ*M* valinomycin, followed by 50 μ*M* CORM-401; **(F)** shows additions of 1 μ*M* valinomycin and then 1 μ*M* CCCP. Results are representative of three independent biological repeats, where net changes in fluorescence were equivalent across all repeats. Fluorescence changes (ΔF) are expressed as arbitrary units (AU). CCCP, carbonylcyanide *m*-chlorophenylhydrazone.

CCCP and other protonophores (uncouplers) have dissociable protons and permeate membranes either as protonated acids or conjugated bases; they therefore facilitate proton exchange across energy-transducing membranes (43). To extend the understanding of energetic implications of CORM-401 administration, we measured membrane potential in whole cells using DiSC3 (5, 61). DiSC3(5) is a cationic cyanine dye that responds fluorometrically to changes in membrane potential by potential-dependent partition between the cells and the extracellular medium. When a cell or membrane interior becomes negatively charged (polarized), the dye is taken up with consequent fluorescence quenching (61). Depolarization, on the other hand, results in release of the dye and increase in fluorescence. The negatively charged interior of respiring *E. coli* cells led to uptake of the dye and slow fluorescence quenching; to collapse the membrane potential (Δψ) and depolarize the membrane, K^+^ and the K^+^-specific ionophore valinomycin were added (Fig. 8A), whereupon net movement of the dye out of the cells resulted in increased fluorescence. However, a solution of CO (50 μ*M* final concentration) did not significantly alter DiSC3 (5) fluorescence (Fig. 8B). In marked contrast, addition of 50 μ*M* CORM-401 (Fig. 8C) or only 1 μ*M* CCCP (Fig. 8D) led to sustained decreases in fluorescence over 15 min, demonstrating polarization of the membrane. Thus, both CORM-401 and CCCP stimulate respiration (Fig. 7) and increase polarization of the membrane (Fig. 8) (see the Discussion section). To determine whether membrane polarization was mediated by potassium flux across the membrane, the potassium gradient was first collapsed by valinomycin (Fig. 8E), followed by additions of CORM-401 (Fig. 8E), or valinomycin followed by CCCP (Fig. 8F). Addition of valinomycin caused a net movement of DiSC3 (5) out of the cell, as in Figure 8A, but when CORM-401 or CCCP was then added, polarization of the membrane was evident from the drop in fluorescence. In the case of CCCP, dramatic polarization was observed (an initial fluorescence decrease) as the collapse of the potassium charge gradient no longer impedes proton movement. The slower subsequent rise in fluorescence is attributed to a slow H^+^ leak after initial hyperpolarization by CCCP. Therefore, polarization caused by CORM-401 and CCCP is potassium independent, suggesting involvement of H^+^ or another cation.

#### Genes involved in potassium and general ion homeostasis and osmolarity are perturbed in response to CORM-401

**FIG. 9.**
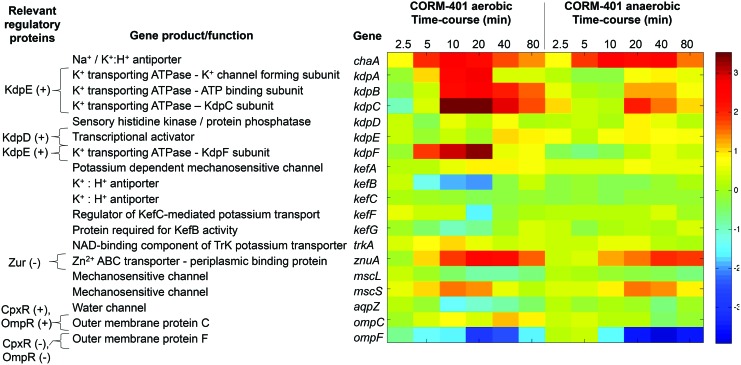
**Differential expression of gene involved in osmoregulation.** The heat map quantifies the changes elicited in selected genes both aerobically and anaerobically in response to 67 μ*M* CORM-401. The heat scale at the *right* is expressed as the natural logarithm of the fold change. To see this illustration in color, the reader is referred to the web version of this article at www.liebertpub.com/ars

In accord with the observed respiratory stimulation and membrane polarization elicited by CORM-401 (above), transcriptomic studies highlighted numerous genes involved in the transport of potassium and zinc and genes involved in osmoregulation and ion homeostasis (Fig. 9). The KdpFABC complex is a multisubunit ATP-driven potassium pump (24). All four genes encoding the membrane transporter were upregulated within 5–10 min after CORM-401 treatment under aerobic conditions (Fig. 9). The *kdp* genes are expressed when K^+^ levels in the cell become limited (33); this expression is regulated by KdpD (sensor kinase) and KdpE (response regulator) comprising a two-component regulatory system. However, measurements of membrane polarization in the presence of valinomycin (Fig. 8) show that CORM-401-elicited polarization is independent of potassium gradients, and so it is likely that the changes in *kdp* gene expression reflect global osmotic changes (30, 34) and/or the physicochemical state of the membrane, as induced by ethanol, procaine, and others (66). In addition to this high-affinity potassium transport system, *chaA*, encoding a K^+^/Na^+^:H^+^ antiporter, was also transiently upregulated both aerobically and anaerobically (Fig. 9). *E. coli* possesses two other K^+^ transport systems, namely Kup and Trk, which exhibit a high transport velocity, but low affinity, and these are constitutively expressed, as evidenced by the invariant levels of *trkA* expression (Fig. 9).

Two possible Ca^2+^ transporters have been identified in *E. coli*, including a Ca^2+^/H^+^ exchanger, ChaA, but it appears not to be a major Ca^2+^ efflux pathway (42); the *chaA* gene was upregulated by CORM-401 after 5 min both aerobically and anaerobically, further implicating osmotic imbalance (Fig. 9). ChaA has also been identified as a K^+^/H^+^ antiporter with roles in potassium homeostasis (54).

Other systems involved in osmoregulation were perturbed by CORM-401. Mechanosensitive channels are membrane transporters that respond to changes in cellular osmotic pressure (65). MscS is a homoheptameric archetypal member of a diverse superfamily of mechanosensitive channels. It possesses a large water-filled cytoplasmic domain, is involved in selectivity, and may function as a cytoplasmic osmometer (47). In response to CORM-401, *mscS* was up to fivefold upregulated both aerobically and anaerobically (Fig. 9), suggesting osmotic stress, perhaps due to uptake of the CORM.

We observed striking downregulation (to undetectable levels; Supplementary Fig. S3B) of the *ompF* gene encoding a large trimeric membrane permeability channel. The expression of this porin and of the other major porin OmpC is exquisitely regulated (45). Because noxious agents such as antibiotics diffuse more readily through the larger OmpF channel, its increased production also facilitates entry of nutrients. Environmental osmotic status is sensed by the EnvZ component of the EnvZ-OmpR two-component system. High osmolarity activates OmpR, and *ompC* expression is increased and *ompF* expression decreases. The present data thus point to bacterial sensing of increased osmotic pressure. The OmpC and OmpF channels appear differentially regulated to tackle changes in osmotic pressure; OmpF may be downregulated to avoid mass movement of unwanted solutes into the cell, including the CORM-401 compound itself, while expression of the smaller channel, OmpC, allows finer control of solute movement. Other systems that control turgor pressure include the aquaporin *aqpZ*, a water channel that allows the bidirectional movement of water in response to osmotic stress (62). This channel was slightly downregulated in aerobic conditions in response to CORM-401 (Fig. 9).

Expression of a zinc-binding subunit of a zinc transporter (*znuA*) (51) was also upregulated by 11-fold after 20 min aerobically (Fig. 9); anaerobically, a delayed rise peaked at sevenfold upregulation after 40 min.

#### CORM-401 leads to changes in cellular potassium and zinc levels

**FIG. 10.**
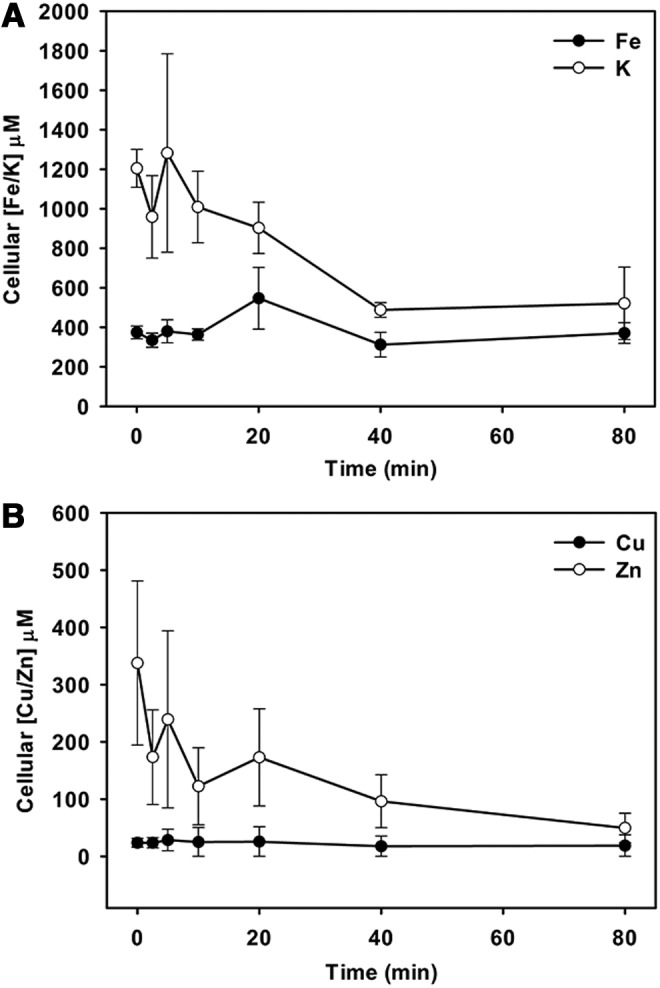
**Potassium and zinc levels fall after CORM-401 addition.** *E. coli* cells were grown to the exponential phase in Evans medium supplemented with 20 m*M* glucose. Samples were taken immediately before (*t* = 0) and at time intervals after 67 μ*M* CORM-401 addition (final concentration). Cell pellets were analyzed using ICP-MS. **(A)** Intracellular potassium and iron levels; potassium falls significantly between *t* = 0 and *t* = 80 (*p* = 0.03). **(B)** Zinc and copper levels; zinc levels fall between *t* = 0 and *t* = 80. *n* = 3 ± SEM. ICP-MS, inductively coupled plasma mass spectrometry.

Informed by the transcriptomic changes that indicated perturbation of metal ion homeostasis or osmolarity and the demonstration that CORM-401 leads to membrane polarization, we measured potassium fluxes using spheroplast swelling experiments. Potassium movements with consequent swelling in the presence of CORMs have already been shown with CORM-3 (73), but CORM-401 did not induce spheroplast swelling in the presence of iso-osmotic potassium nitrate/potassium nitrite (data not shown). Although passive potassium movements appear not to be invoked by CORM-401, other data (above; upregulation of *kdp* and downregulation of potassium efflux machinery) suggested that bacteria experienced potassium limitation. We therefore assessed the levels of total intracellular potassium and trace metals in the presence of CORM-401 using ICP-MS. Figure 10 shows that 67 μ*M* CORM-401 decreased the intracellular concentration of potassium and zinc by about threefold over 40 min. There were no measurable changes of copper or iron levels in response to CORM-401 addition, nor were genes implicated in the metabolism of these altered ions.

#### CORM-401 causes induction of the Cpx and Bae regulons, altering the expression of Spy and CpxP proteins, but without measurable membrane damage to cells

**FIG. 11.**
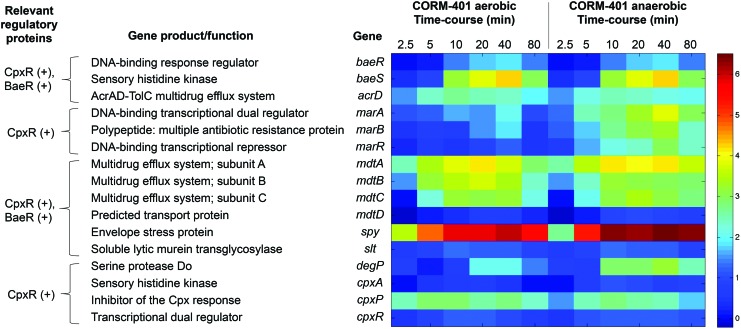
D**ifferential expression of genes implicated in general stress responses, metal ion stress, and cell envelope stress.** The color-scale bar shows mean fold changes in individual genes of WT *E. coli* both aerobically and anaerobically in response to 67 μ*M* CORM-401. The heat scale at the *right* is expressed as the natural logarithm of the fold change. To see this illustration in color, the reader is referred to the web version of this article at www.liebertpub.com/ars

The Cpx system in *E. coli* protects the membrane during stress (55), such as high osmolarity, leading to the management of cellular processes that include motility, chemotaxis (13), and biofilm formation (14). Exposure of cells to CORM-401 upregulated numerous genes under the transcriptional control of Bae/Cpx (Fig. 11). Genes encoding the multidrug efflux system *(mdtA-D)* were more than 60-fold upregulated under both aerobic and anaerobic conditions (Supplementary Fig. S3C). Upregulation of the Cpx response by CORM-2 (46) and involvement of the Cpx and Bae systems in response to CORM-3 have been noted previously (9, 38). The most dramatic perturbation was that of the periplasmic chaperone Spy, which was upregulated by >400-fold aerobically and >600-fold anaerobically (Supplementary Fig. S3C); CORM-3 elicits similar changes (9, 38).

**FIG. 12.**
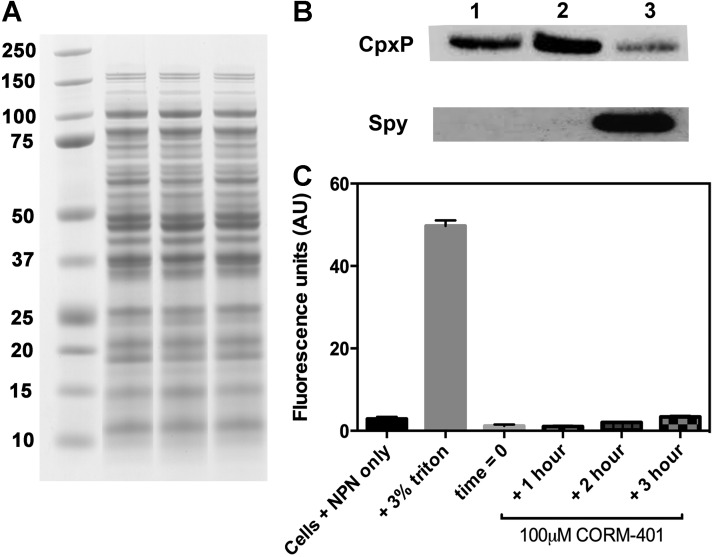
**CORM-401 leads to upregulation of cellular stress responses, but does not perturb the outer membrane of** ***E. coli*. (A)** Coomassie-stained SDS gel of soluble fractions used in Western blotting illustrates equal loading of protein. Molecular mass markers (kDa) are shown on the *left*. **(B)** A typical Western blot of subcellular fractions is shown in the absence (lane 1) and presence of 67 μ*M* DTC/MnSO_4_ (lane 2) or 67 μ*M* CORM-401 (lane 3) for 2 h with anti-CpxP and anti-Spy antibodies. Data shown are representative of three biological replicates. **(C)** Cells were resuspended in PBS, then exposed to NPN alone (*black bar*), NPN +3% triton (positive control) (*gray bar*), or 100 μ*M* CORM-401 for increasing time periods (as labeled). All concentrations given are final concentrations in the fluorescence cuvette. *n* = 3 ± SEM.

To determine whether the increased transcripts reflected protein levels, Western blot assays were carried out using antiserum samples to two key players in the response, Spy and CpxP (74). Spy was detected in periplasmic fractions of wild-type cells and CpxP was measured in total soluble (cytoplasmic and periplasmic) fractions. Addition of 67 μ*M* CORM-401 significantly increased Spy protein levels after 2 h (Fig. 12B, lane 3), whereas incubation with the control compounds MnSO_4_ and DTC did not raise Spy to detectable levels (Fig. 12B, lane 2); the control in the absence of CORM showed no detectable Spy levels (Fig. 12B, lane 1). Levels of the periplasmic chaperone CpxP were clearly detected in the absence of CORM (Fig. 12B, lane 1) and also on incubation with control compounds (Fig. 12B, lane 2), but a significant decrease in CpxP abundance was seen after 2 h of CORM-401 treatment (lane 3). The CORM-induced decrease in CpxP is consistent with literature on the Cpx response (55); since CpxP is a negative regulator, its levels are lowered to achieve the Cpx response evident in Figure 11.

CORM-3 upregulates *spy* expression and increases Spy levels due to membrane damage (74). The effects of CORM-401 on bacterial outer membranes (OMs) were therefore assayed using *N*-phenyl-1-napthylamine (NPN), a membrane-impermeable fluorophore that increases fluorescence in a hydrophobic environment (61). Thus, when the bacterial membrane becomes perturbed (*e.g*., by an antibiotic or CORM-401), the dye partitions into the outer membrane, leading to an increase in fluorescence. Interestingly, the addition of CORM-401 to cells in the presence of NPN showed no increase in membrane damage, even after several hours (Fig. 12C). The detergent Triton X-100 was used as a positive control (Fig. 12C). Thus, the action of CORM-401 is distinct from CORM-3: the upregulation of Spy protein and transcript levels by CORM-401 appears indirect and due to perturbation of cellular osmotic balance and metal ion homeostasis.

The *spy* gene is positively regulated by phosphorylated CpxR, whereas several motility genes are negatively controlled (13). Indeed, Figure 4 shows that on incubating bacteria with CORM-401 for 5 or more min, the expression of motility genes was decreased. However, within the first 5 min of exposure to CORM-401 or after 80 min of incubation, a majority of motility genes were upregulated (Fig. 2). To resolve this, we measured motility (swarming) as before (38). After 48 h of incubation, the mean colony diameters were measured as 12.2 (±2.2 SD) mm for control colonies, whereas cells grown with 67 μ*M* CORM-401 (as used for Fig. 4) had an increased colony diameter of 24.2 (±0.8 SD) mm (data not shown). However, control compounds Mn(II) sulfate and sarcosine DTC increased motility further (colony diameter of 32.3 ± 2.3 SD mm). Student's *t*-test revealed that the difference between these data sets was highly significant (*p*-value <0.001). We conclude that bacterial motility is not modulated by intact CORM-401.

#### A CORM-401 growth screen of several pathogens isolated from clinical infections shows varying susceptibility

**FIG. 13.**
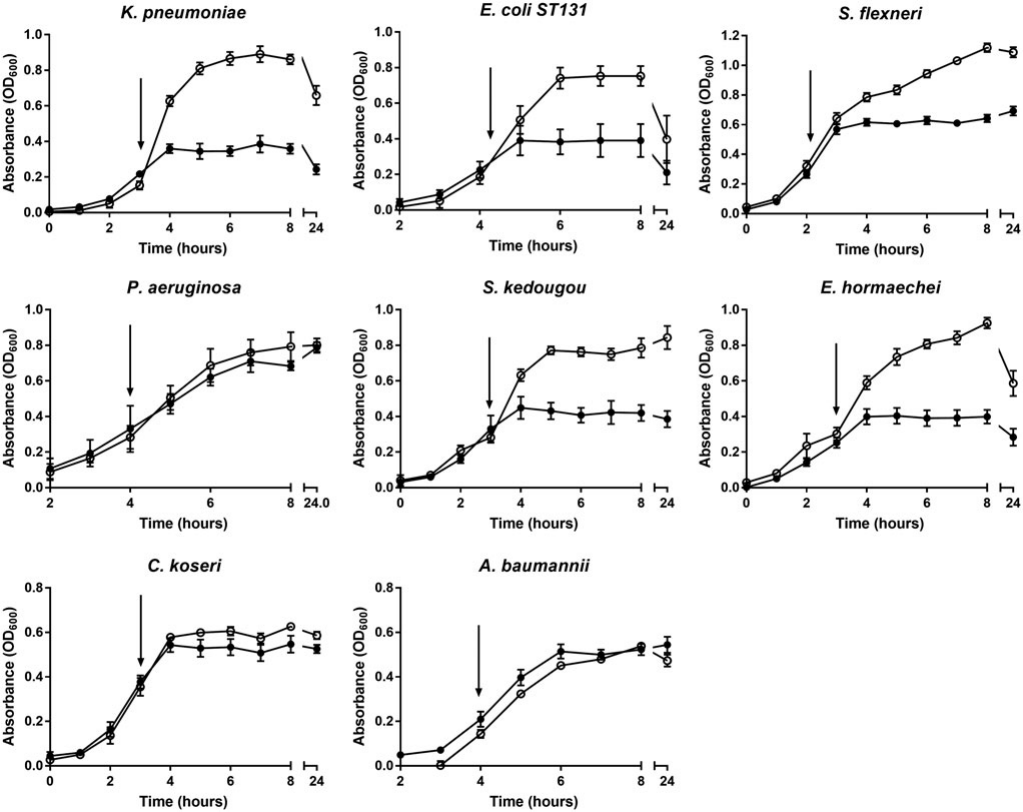
**A growth screen of pathogens isolated from clinical infections shows varying susceptibility to CORM-401.** Strains were grown in Evans medium with glucose to the early exponential phase before the addition of 500 μ*M* CORM-401 (*closed circles*, addition indicated by *arrows*) or the equivalent volume of PBS (*open circles*). Growth was monitored throughout, *n* = 3 ± SD.

To broaden the significance of this study, eight clinical isolates of pathogenic bacteria were tested for their sensitivity to the title compound. *E. coli* EC958 is a multidrug-resistant O25b:H4 clinical pathogen (68). The genome encodes numerous putative virulence factors, including siderophore receptors and autotransporters, and bears genes conferring resistance to ciprofloxacin and other antibiotics (68). We also tested clinical isolates of *Klebsiella pneumoniae*, *Shigella flexneri*, *Pseudomonas aeruginosa*, *Salmonella enterica* serovar Kedougou, *Enterobacter hormaechei*, *Citrobacter koseri*, and *Acinetobacter baumannii*. Cultures were grown to early exponential phase before the addition of CORM-401 (final concentration, 500 μ*M*). Growth was monitored until the stationary phase was reached in cultures not exposed to the compound (Fig. 13). Addition of the metal carbonyl caused a complete arrest in growth for *K. pneumoniae*, *E. coli* EC958, *S. flexneri*, *S.* Kedougou, and *E. hormaechei* cultures; however, growth of *P. aeruginosa*, *C. koseri*, and *A. baumannii* was unperturbed by the compound.

#### CORM-401 toxicity in combination with antibiotics

A promising approach to the rise of antibiotic-resistant bacteria, compounded by slow emergence of new drugs, is the use of nonantibiotic compounds to complement existing antibiotics (15, 20). While antibiotics generally target specific cellular processes, such as DNA replication and synthesis of cell walls and proteins, CORMs have numerous targets, as evidenced here. Thus, we tested CORM-401 interactions with two antibiotics, cefotaxime and trimethoprim, against strain MG1655. Addition of 100 μ*M* CORM-401 with antibiotics at sublethal doses led to a greater statistically significant reduction of growth compared with either the antibiotic or CORM-401 alone (Supplementary Fig. S4A, B). However, viability assays with cefotaxime (Supplementary Fig. S4C) or trimethoprim (Supplementary Fig. S4D) showed that each antibiotic and CORM-401 significantly reduced viable cell numbers, even when used individually. To quantify these interactions, we used checkerboard dilution plates and calculated the fractional inhibitory concentrations (FICs) for each combination. For cefotaxime and trimethoprim (Supplementary Fig. S4) as well as for novobiocin and cefotaxime, FIC values were between 0.54 and 0.91 (Table 1), indicative of no interaction (48) between the two antimicrobial compounds.

**Table 1.** Interactions of CORM-401 with Antibiotics Having Different Modes of Action

*Antibiotic*	*FIC_antibiotic_*^a^	*FIC_CORM_*^b^	*∑FIC = FIC_antibiotic_ + FIC_CORM_*	*CORM/antibiotic interaction*
*Escherichia coli* strain MG1655				
Doxycycline	0.25	0.66	0.91	No interaction
Trimethoprim	0.12	0.42	0.54	No interaction
Novobiocin	0.33	0.33	0.66	No interaction
Cefotaxime	0.12	0.66	0.78	No interaction
*E. coli* strain EC958				
Colistin	0.5	0.06	0.56	No interaction
Doxycycline	0.5	0.13	0.63	No interaction
Gentamicin	1.0	0.03	1.03	No interaction

FIC values (calculated as described in the Materials and Methods section) are shown for CORM-401 in combination with the selected antibiotics. MIC values are expressed as μg mL^−1^. The description of the interaction follows the recommendation of odds (48).

^a^ MIC of antibiotic in combination/MIC of antibiotic alone.

^b^ MIC of CORM in combination/MIC of CORM alone.

CORM, carbon monoxide-releasing molecule; FIC, fractional inhibitory concentration; MIC, minimal inhibitory concentration.

The World Health Organization, in its recent global priority list of antibiotic-resistant bacteria, identifies antibiotic-resistant Enterobacteriaceae, including *E. coli*, as being among the most critical (71). Because of the broad antibiotic resistance of strain EC958, we selected colistin (a drug of last resort), doxycycline, and gentamicin. In each case, FIC values were between 0.56 and 1.03 (Table 1), again indicative of no interaction between the two antimicrobial compounds. In conclusion, the bactericidal and bacteriostatic activities of CORM-401 are not synergistic with antibiotic action.

## Discussion

CORMs, originally devised to mimic the beneficial antioxidant, anti-inflammatory, and cytoprotective benefits of CO, serendipitously proved to be effective antimicrobial agents. In particular, the ruthenium compounds CORM-2 and CORM-3 are highly effective antimicrobials (12, 73), but the basis of their efficacy remains unresolved. CO release appears to be a minor contributor to microbial toxicity and attention has turned to the role of Ru (63).

Interestingly, a manganese photoactivated CORM, ([Mn(CO)_3_(tpa-κ^3^*N*)]^+^), is less effective than the Ru complexes (41), unless combined with hydrogen peroxide (67). The present compound, CORM-401, is also a manganese complex, on which few studies are published. In previous work, it released 2.1–3 mol CO in the myoglobin assay (16), in line with our determination of 2.4–2.5 mol in minimal growth medium or phosphate buffer and with myoglobin at a fourfold excess over CORM-401. In mammalian studies, the higher CO yield elicited more effective aortic relaxation and vasodilation than did CORM-A1, which has a similar half-time of CO release, but a lower molar CO yield. CORM-401 reduces inflammation and damage in pig kidneys in a preclinical model of organ donation (3) and reverses the metabolic changes that occur during lipopolysaccharide-induced microglia inflammation (72).

In endothelial cells, there is evidence that the CO released from CORM-401 uncouples mitochondrial respiration and inhibits glycolysis (28) since inactive CORM-401 (iCORM) (actually a mix of MnSO_4_ and the CORM-401 ligand DTC) did not induce these effects. However, even 300 μ*M* CORM-401 elicited only twofold elevation in the oxygen consumption rate, whereas only 1 μ*M* CCCP gave greater stimulation (28). CORM-401 therefore exerts relatively weak, but clear uncoupler-like, activity in mammalian cells, a result confirmed here in bacteria (Fig. 7C). In endothelial cells, CORM-401 also reactivated mitoBK_Ca_ channels after blockage with paxilline (28). The mechanistic basis of most of these effects on mitochondria is unclear, but it is assumed that at the highest CORM concentrations, the decrease in respiration rate is due to inhibition of cytochrome oxidase activity (28). Activation of mitochondrial uncoupling proteins has been proposed to explain CORM-3-uncoupled metabolism in cardiomyocytes (36).

**FIG. 14.**
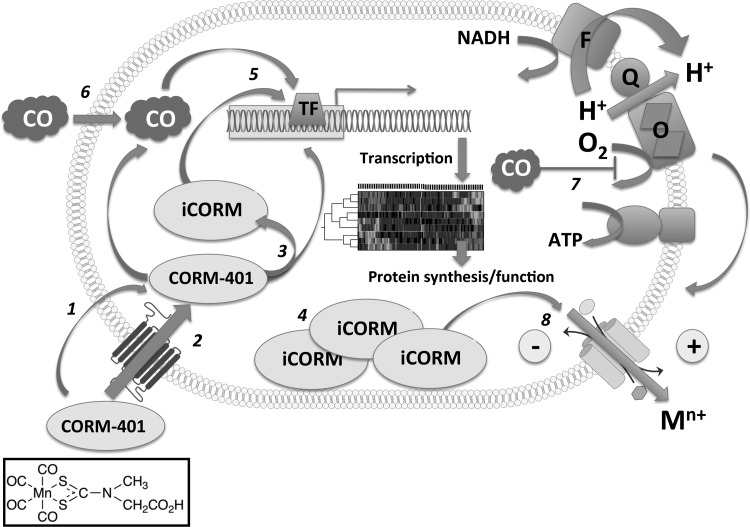
S**chematic diagram of a hypothesis for the antimicrobial effects of CORM-401.** CORM-401 (structure shown at *bottom left*), administered extracellularly, is transported inward to the cytoplasm either by **(1)** diffusion or **(2)** by an unidentified transporter. Within the cell, **(3)** CO is released leaving a metal co-ligand fragment (iCORM). CORM accumulates to **(4)** very high levels, perhaps as a result of the maintenance of a concentration gradient, following conversion of the CORM (outside) to iCORM (inside). CORM-401 and/or its breakdown products elicit **(5)** major transcriptional changes, reflected in altered protein synthesis/function. CO, whether released from CORM within cells or **(6)** following gas diffusion from outside, **(7)** binds to oxidases (O) and partly inhibits aerobic NADH oxidation *via* flavins (F) and quinones (Q). This perturbs the protonmotive force, which is matched by enhanced respiration leading to membrane polarization, which in turn drives further uptake. **(8)** Translocation of cations (M^n+^) such as K^+^ or Zn(II) is shown.

Our data suggest an alternative mode of action for growth inhibition of *E. coli* (Fig. 1) and many other bacteria (Fig. 14) and the myriad effects of CORM-401. First, the released CO does target terminal oxidases (Fig. 7A) leading to slight reductions in oxygen consumption rates (Fig. 7B, D), but the stimulatory effects on respiration are much more significant (Fig. 7C). In-depth transcriptomic and modeling studies (Figs. 4–6) confirm effects on respiratory gene regulation. More striking are the elevations in intracellular Mn concentrations (and thus the CORM, Fig. 3). We suggest that buildup of the metal co-ligand fragment in the cytoplasm to millimolar concentrations leads to disruption of charge (Fig. 8) and ion balance (Fig. 10) and the perception by the cell that it is undergoing osmotic stress (Figs. 9, 11, 12). These ideas are summarized in Figure 14. The mechanism of polarization caused by CORM-401 is unclear, but is independent of potassium gradients (Fig. 8) and presumably caused by perturbation of other ionic balances across the membrane. In mammalian systems also, the CORM-401-enhanced resistance of cardiomyocytes to oxidative stress may be, at least in part, due to the manganese center (31). However, in contrast to (28), we cannot attribute the effects on membrane polarization to the released CO since a CO gas solution is without effect on membrane potential (Fig. 8B). Our proposals that CORM-401 toxicity is not dependent on respiratory inhibition are in accord with other studies, notably the finding that CORM-3 is toxic to bacteria even in the absence of a heme target (74).

CORM-401 stimulates mitochondrial (28) and bacterial [(73); this work] respiration, a response mimicked by the classical uncoupler CCCP (Fig. 7C). However, the fact that CORM-401, like CCCP, caused polarization of the membrane (Fig. 8) suggests that this is compensated by enhanced respiration. That bacteria remain viable after CCCP treatment and can mount such a respiratory response is evidenced by the fact that neither 100 μ*M* CORM-401 (this work, Fig. 2) nor 1 μ*M* CCCP significantly inhibits growth and respiration remains responsive to imposed membrane conditions. Indeed, 1 μ*M* CCCP elicits only a 2% decrease in swimming speed of *E. coli* (4).

CORM-401 has a net negative charge in solution, yet is rapidly accumulated to millimolar levels in the cell (Fig. 3A). The charged metal species produced when the compound releases CO might also affect charge separation across the membrane. It is expected that when CO groups are released from CORM-401, the metal in the residual co-ligand fragment will become oxidized giving an Mn(II) species. It is therefore interesting that upon CORM-401 addition, cells become polarized; this may be due to perturbation of charge separation across the membrane or by accumulation of compatible solutes, with little or no negative charge, to balance the influx of positive charge (75). In addition to polarization, loss of K^+^ and Zn(II) from the cell (Fig. 10) may result from accumulation of charge. The mechanosensitive channel, MscS, which is upregulated in response to osmotic pressure, was also upregulated (Fig. 9). We suggest that a contributing factor to the action of CORM-401 is generation of high osmotic pressure upon extreme accumulation of the compound (Fig. 3). Although cellular envelope stress responses are upregulated at the transcriptomic and protein levels, and transport systems for metal ions and water are differentially expressed in response to CORM-401 (Fig. 8), CORM-401, unlike CORM-3, has little effect on the cell membrane as assessed by NPN fluorescence.

In this study, we demonstrate for the first time that CORM-401 also has broad-spectrum antimicrobial activities. However, CORM-401 exhibits toxicity toward eukaryotic cells: at 100 μ*M*, a 25% decrease in viability of RAW264.7 cells was noted (7), whereas this concentration had little effect on *E. coli* viability up to 4 h (Fig. 2C, D). CORM-401 toxicity is dependent on the carbon source used in the growth of the bacterium, probably reflecting differences in uptake (Fig. 3). Although CO released from CORM-3 and ([Mn(CO)_3_(tpa-κ^3^*N*)]^+^) binds cellular targets such as respiratory heme oxidases, recent data suggest that CORMs display numerous other modes of toxicity.

The multiple modes of action of CORM-401 and other CORMs are clearly distinct from the focused effects of most antibiotics, suggesting that these compounds, even before we comprehensively understand their targets, should be valuable as antimicrobial agents that could enhance antibiotic sensitivities. Indeed, CORM-401 enhances, without interaction, the efficacy of four antibiotics on *E. coli* (Table 1). It is notable that CO gas neither potentiates the toxicity of antibiotics nor protects from their effects (69).

The different sensitivities of clinical isolates to CORM-401 probably reflect different metabolism and import/export mechanisms. The resistance of *P. aeruginosa* to CORM-401 appears contrary to the results of previous research using CORM-2 (40). However, *P. aeruginosa* and *A. baumannii* express multiple efflux pumps, making them resistant to numerous antibiotics (19, 64). The hypothesis that these strains fail to accumulate CORM-401 intracellularly might be tested by analyses of manganese contents and comparison of sensitive and resistant strains. Resistance to CORM-401 may also reflect the ability of resistant bacteria to produce and excrete mucoid substances forming a peripheral capsule around the pathogens, hindering access of the metal carbonyl compound. Capsule production decreases sensitivity to antibiotics and renders pathogens resistant to phagocytosis by macrophages and to the toxic effects of free radical species (21, 22). The potential for CORMs to act as novel antimicrobials against other clinical isolates is indicated by the sensitivity of numerous bacterial species, including several members of the family Enterobacteriaceae. For example, the sensitivity of the urinary tract and bloodstream pathogen *E. coli* EC958 to CORM-401 suggests that exploration of these CO releasers as potential anti-UTI drugs either alone or with current antibiotics is a realistic possibility.

An ongoing challenge is to understand the bases of the antimicrobial actions of CORMs and use this knowledge to devise scaffolds with increased activity. Although CORM-401 is a less effective antimicrobial agent than CORMs-2 or −3, such data contribute to our understanding of the effects of CORMs in general and the development of future CORMs as antimicrobials; greater consideration of the effect of the metal center could lead to the generation of more potent and biologically compatible CORMs.

## Materials and Methods

### *E. coli* strains and batch growth conditions

*E. coli* K12 derivative MG1655 was used as a model organism for this study; other pathogenic clinical isolates were tested where indicated. All strains were grown in Evans medium with glucose or succinate (20 m*M* each) as the carbon source (27). For *S. flexneri* and *A. baumannii*, MEM amino acid (50 × –Sigma-Aldrich) solution was added to facilitate growth. Unless stated, cells were grown to the mid-exponential phase (OD_600_ ∼ 0.5–0.6, 50–60 Klett units) before adding CORM-401 or other compounds. Growth was monitored using a Klett-Summerson colorimeter using a red filter in 250-mL conical flasks fitted with side arms or spectrophotometrically at 600 nm. Note that bacterial cultures begin growth at different intervals after inoculation, so averaging several similar experiments on a time basis is inappropriate. Growth curves generally show one experiment representative of three or more that showed similar kinetics and growth rates.

### Chemostat growth conditions

For continuous culture, cells were grown in an Infors Multifors bioreactor (total volume 200 mL) adapted to fit a Labfors-3 fermenter base unit. Temperature was maintained at 37°C with continuous stirring at 200 rpm; the dilution rate was 0.2 h^−1^. Mass flow controllers allowed gas mixes for aerobic and anaerobic conditions to be maintained by continuous bubbling at 100 mL min^−1^ with air and N_2_ gas (aerobic) or N_2_ gas alone (anaerobic) as before (38).

### CORM-401 and control compounds

CORM-401 was synthesized in the Department of Chemistry, The University of Sheffield, as before (7). Stock solutions (5 m*M*) were prepared fresh daily in phosphate-buffered saline (PBS), pH 7.4. There is no useful iCORM, that is, an inactive compound for control experiments; instead, MnSO_4_ (0.1 *M* stock solution) and sodium dithiocarbamate (DTC, Na[S_2_CN(CH_3_)CH_2_COONa], 10 m*M* stock solution) were combined to give equimolar mixes of the two compounds, as required. Once the Mn in CORM-401 loses CO, Mn(I) will probably be oxidized to Mn(II). Mn(II) is kinetically labile and it is probable that the [O_2_CCH_2_NMeCS_2_]^2−^ ligand dissociates. Others refer to such a mixture as inactive CORM-401 (16), but its physiological and transcriptomic effects have not been evaluated.

### Myoglobin assay for CO release

CO liberated from CORM-401 was assayed using the reaction with ferrous myoglobin (39), but final concentrations of myoglobin and CORM were 15 μ*M* and 3 μ*M*, respectively (to allow equistoichiometric binding to myoglobin of the anticipated 3–4 mol CO released from CORM-401). All assays were carried out at 37°C unless otherwise indicated.

### Subcellular fractionation and metal analyses

Exponential cultures (1 L) were supplemented with 67 μ*M* CORM-401 or, as a control, DTC/MnSO_4_ and incubated at 37°C with shaking at 200 rpm for 90 min before centrifugation (10 min, 12,000 *g*). The supernatant was retained for analysis and the cell pellet was resuspended in ∼6 ml of 200 m*M* PBS (pH 7.0) before sonication on ice (MSE Soniprep, 16 μm, 6 × 15 s bursts). Following centrifugation (20 min, 20,000 *g*), the pellet comprising cell debris was discarded. From the supernatant, membrane and cytoplasmic fractions were isolated by ultracentrifugation (60 min, 160,000 *g*) using centrifuge tubes prewashed in concentrated nitric acid to remove trace metals. Samples of genomic DNA were isolated from independently grown cultures under identical growth conditions using the Wizard^®^ Genomic DNA Purification Kit and the manufacturer's instructions. Metals were assayed in all fractions by inductively coupled plasma mass spectrometry, as described below.

### Assay of CO binding to cellular heme proteins

Spectra were recorded with an Olis RSM1000 dual-beam rapid scanning monochromator (On-Line Instrument Systems) fitted with a clarity accessory as before (56). Cells were resuspended in PBS to an OD ∼55 and CORM-401 was added to a final concentration of 100 μ*M*. Scans were taken at intervals up to 15 min after addition of CORM-401.

### Assays of cellular respiration

In closed electrode experiments, oxygen consumption was measured using a Clark-type electrode (27) (Rank Brothers, Bottisham, Cambridge, United Kingdom). The chamber contents (2 mL) were stirred at 37°C, while the top was sealed with a close-fitting lid that permitted addition *via* microsyringes of reagents. For prolonged measurements of respiration, a custom electrode apparatus open to the atmosphere based on published designs (11) was used. The Perspex cell (working volume 4 mL) was maintained at 37°C by circulating water, the cell being constructed of stainless steel to aid temperature equilibration. The two-bladed stirring impeller (diameter 14 mm) was mounted on a stainless steel shaft that was stirred using an overhead stirrer (IKA^®^-Werke Eurostar power control-visc P4) to maintain a stable vortex and absolute constancy of rotational speed and therefore of transfer of air from the atmosphere to the stirred sample. The rate of oxygen diffusion from the atmosphere to the sample was expressed as *K_L_a*, measured as in (52). The additions made were of CORM-401 (stock solution 5 m*M*), carbonyl cyanide *m*-chlorophenylhydrazone (CCCP; stock solution 10 m*M*), or KCN (stock solution 15 m*M*).

### Assays of membrane potential

Cells were washed, resuspended in 5 m*M* HEPES buffer to a final OD_600_ of 0.6, and incubated with 0.1 *M* KCl and 10 m*M* glucose before treatment for ∼5–10 min with 0.4 μ*M* DiSC3(5) in a 3-mL quartz cuvette. Fluorescence was measured as before using a Hitachi F-2500 fluorescence spectrophotometer.

### Assays of cell motility

Motility assays were performed as previously described (38). Briefly, *E. coli* MG1655 cells were aerobically grown to the stationary phase in glucose-supplemented Evans medium and 10 μL of the liquid culture was spotted onto 0.3% (w/v) LB/agar plates containing 67 μ*M* CORM-401 or the control compounds DTC/MnSO_4_. Plates were incubated for 48 h at 30°C and colony diameters measured. Each of 3 biological replicates comprised five technical determinations.

### Transcriptomic analysis and statistical modeling

These procedures were conducted as before (38, 69, 74) except that samples were taken from chemostat cultures of glucose-grown cells immediately before CORM-401 addition and at intervals thereafter. In brief, extracted RNA was labeled with Cy-3 or Cy-5-dCTP and hybridized to Agilent arrays that were scanned using an Agilent DNA Surescan Microarray scanner with subsequent feature extraction and data analysis using GeneSpring GX v7.3. Arbitrary values of ≥2-fold (*i.e*., ≥2-fold increased expression) or ≤0.5-fold (*i.e*., ≤2-fold decreased expression) were chosen to identify genes with significantly altered expression. Functional category gene lists were created using KEGG (Kyoto Encyclopedia of Genes and Genomes) (69). Where available, regulatory proteins for each gene were identified using EcoCyc. Modeling of TF activities using TFInfer (2, 23, 58) and measuring similarity in TF activities between two different conditions were performed as described in (38).

### Metal analyses

Culture samples (20 mL) were taken before and after addition of 67 μ*M* or 500 μ*M* CORM-401 and assayed for metal content as before (38). Intracellular metal concentrations were calculated using literature values for cell volume (23). In control experiments, cultures were treated instead with 500 μ*M* MnSO_4_ ±67 μ*M* DTC or a saturated solution of CO giving a final concentration of 500 μ*M* CO. To estimate total numbers of manganese atoms per cell, the data of Outten and O'Halloran (49) were used.

### OM permeabilization assays

Outer membrane (OM) permeability of CORM-401 was assayed using 1-N-phenylnaphthylamine (NPN) (61) at a final concentration of 1 μ*M*. Cells were grown to the exponential phase (OD_600_ of 0.6), pelleted, then washed, and resuspended in PBS. The final cell suspension was adjusted to an OD_600_ of ∼0.5. Fluorescence was measured (λ_ex_ = 340 nm, λ_em_ = 420 nm) using a Hitachi F-2500 fluorescence spectrophotometer.

### Spheroplasts and osmotic swelling measurements

To measure transmembrane ion fluxes, osmotic swelling was measured by following changes in turbidity at 500 nm following dilution of spheroplasts in isoosmotic (0.25 *M*) salt solutions as before except that EDTA/lysozyme treatment was at 37°C (74).

### Western blotting for Spy and CpxP detection

This was done as before (74). In brief, CORM-401 or DTC/MnSO_4_ was added to cultures to a final concentration of 67 μ*M* and incubated for 2 h. For Spy quantitation, periplasmic fractions were isolated using the Tris/sucrose/EDTA (TSE) method (53). For CpxP, soluble fractions were made after cell breakage by sonication, differential centrifugation, followed by reduction with 200 m*M* dithiothreitol, and separation by SDS-PAGE. Proteins were blotted using primary rabbit anti-Spy/CpxP antibodies at 1:25,000/1:50,000 dilutions, respectively. Anti-rabbit secondary antibodies were incubated at a concentration of 1:50,000 before detection using the ECL-Plus Western system (Amersham).

### Growth and viability studies of CORM-401 in conjunction with antibiotics

Bacteria were grown in Evans medium with 20 m*M* glucose as a carbon source until 0.3 OD_600_ was reached; CORM-401 (100 μ*M*) and/or trimethoprim (1.0 μg/mL) or cefotaxime (1.0 μg/mL) were added alone or in combination. The interactions of CORM-401 with antibiotics were determined using a broth microdilution checkerboard assay (44). Briefly, bacteria were diluted in Evans medium to reach an OD of around 0.3 (∼5 × 10^5^ cfu/mL) and 200 μL of the suspension pipetted into each well of a 96-well plate. The concentrations used for wild-type MG1655 were CORM-401 (100–600 μ*M*), trimethoprim (1–16 μg/mL), and cefotaxime (1.0–32 μg/mL). For strain EC958, concentrations were CORM-401 (37.5–600 μ*M*), colistin (0.25–8 μg/mL), doxycycline (6–96 μg/mL), and gentamicin (0.125–4 μg/mL) added alone or in combination with CORM-401. Cultures were incubated at 37°C with shaking for 24 h using a Tecan Sunrise plate reader. The concentrations tested were up to four 2-fold dilutions lower than the minimal inhibitory concentration (MIC) and, where possible, two 2-fold dilutions higher than the MIC. The MIC was considered as the lowest concentration of the agent alone or combined with CORM-401 that inhibited growth. Fractional inhibitory concentration index (FICI) (44) was calculated to determine drug interaction and interpreted as follows:

FICI of two-drug combination = FIC_A_ + FIC_B_, where FIC_A_ is the MIC of drug A in combination with CORM-401/MIC of drug A alone and FIC_B_ is the MIC of drug B in combination with CORM-401/MIC of drug B alone. The results indicate synergy when the calculated FICI ≤0.5, no interaction when FICI >0.5–4, and antagonism when the FICI >4 (48).

### Statistical analysis

All data are expressed as the mean ± SEM, unless otherwise stated. The comparison of the means was performed using Student's *t*-test for two groups of data. For comparison of data across more than two groups, ANOVA, followed by Bonferroni's multiple comparison tests, was used. When *p* < 0.05, data were considered significantly different.

## Supplementary Material

Supplemental data

## Abbreviations Used

CCCP: carbonylcyanide *m*-chlorophenylhydrazone
CORM: carbon monoxide-releasing molecule
DTC: dithiocarbamate, Na[S_2_CN(CH_3_)CH_2_COONa]
FIC: fractional inhibitory concentration
FICI: fractional inhibitory concentration index
ICP-MS: inductively coupled plasma mass spectrometry
MIC: minimal inhibitory concentration
NPN: *N*-phenyl-1-napthylamine
OM: outer membrane
PBS: phosphate-buffered saline
PTS: phosphotransferase system
TF: transcription factor
