# Supplementary material for: The Broad-Spectrum Antimicrobial Potential of [Mn(CO)4(S2CNMe(CH2CO2H))], a Water-Soluble CO-Releasing Molecule (CORM-401): Intracellular Accumulation, Transcriptomic and Statistical Analyses, and Membrane Polarization
Source: Antioxid Redox Signal. 2018 May 10;28(14):1286–308. doi: 10.1089/ars.2017.7239 (PMC5905950; doi:10.1089/ars.2017.7239)
Supplement: Supplemental data [file Supp_Data.pdf]

Supplementary Data

Supplementary Table S1. List of the Thirty Most Highly Regulated Genes, Both Aerobically and Anaerobically

		Time (min, after CORM-401 addition)					
	Gene	2.5	5	10	20	40	80
Aerobic	*spy*	34.3	124.4	357.1	330.3	420.8	250.2
	uhpT	3.3	18.0	76.2	124.2	216.7	142.8
	mdtA	12.4	29.6	46.3	63.2	41.8	17.1
	baeS	1.4	2.3	25.1	45.4	67.4	19.7
	mdtB	4.4	24.3	32.6	42.5	30.2	10.8
	kdpC	0.4	1.5	32.8	33.8	18.2	5.5
	yhdV	3.1	5.5	7.9	29.0	12.5	5.7
	yebE	6.6	20.7	39.8	28.9	19.7	9.4
	kdpF	0.9	6.2	20.8	28.0	1.8	2.0
	mdtC	1.0	9.8	24.7	22.5	24.2	8.6
	kdpA	0.9	2.9	11.3	15.6	1.5	1.5
	kdpB	0.7	1.3	9.8	13.8	7.5	4.8
	ycfS	3.4	10.6	19.9	13.6	9.8	5.1
	cpxP	12.3	17.2	17.7	13.6	16.8	10.6
	b3914	9.6	17.4	18.0	13.4	15.1	10.3
	cpxP	9.7	17.7	18.4	12.6	16.1	10.6
	ybfA	2.9	5.1	6.1	11.8	7.4	3.0
	htpX	5.1	9.2	12.7	11.7	11.7	7.4
	znuA	1.0	2.6	6.5	11.2	10.0	5.2
	acrD	4.8	10.7	15.2	11.2	10.9	6.9
	chaA	2.2	7.0	14.4	9.8	5.8	4.1
	ybdZ	1.6	4.1	4.1	9.3	2.8	2.1
	metF	0.9	0.0	2.4	9.0	3.7	1.0
	yobB	4.7	9.1	11.9	8.4	6.4	4.9
	ybdZ	1.7	3.8	3.5	8.3	2.0	2.0
	yaaX	2.9	4.2	7.5	8.3	6.3	3.3
	glvC	0.5	1.0	1.0	7.9	1.3	3.3
	entA	0.8	1.8	5.3	7.8	4.7	1.8
	degP	2.2	1.2	1.9	7.5	7.4	4.1
	yedX	2.2	6.0	4.3	7.4	5.0	4.2

		Time (min, after CORM-401 addition)					
	Gene	2.5	5	10	20	40	80
Anaerobic	*spy*	14.3	200.4	585.0	505.5	672.6	556.4
	uhpT	1.1	8.3	89.5	112.6	112.1	106.3
	mdtA	12.4	35.2	63.0	59.9	60.2	39.3
	baeS	0.9	1.1	31.0	38.9	25.2	14.3
	yebE	2.4	17.2	39.1	32.0	28.0	11.6
	mdtC	1.0	7.9	22.4	29.8	21.5	17.0
	marA	3.6	11.4	16.7	28.6	47.8	20.4
	mdtB	1.1	10.4	24.8	23.0	20.8	18.4
	marB	3.3	5.6	13.9	21.7	26.7	10.3
	ycfS	2.1	25.7	42.2	20.1	20.7	6.1
	degP	0.8	2.0	18.9	18.5	23.9	12.0
	htpX	2.4	10.5	16.1	14.5	12.5	7.6
	ykgM	1.5	1.2	2.5	14.3	43.3	40.9
	metB	1.4	1.1	5.6	13.0	2.6	0.8
	cpxP	4.6	11.9	17.0	12.9	11.5	5.9
	b3914	4.1	11.9	17.1	12.4	11.5	5.9
	cpxP	4.7	11.4	13.0	11.7	11.3	6.5
	acrD	1.9	8.9	11.3	11.1	8.2	7.6
	marR	1.8	5.1	8.3	10.9	20.2	10.8
	metF	2.0	1.2	5.9	9.8	1.5	0.7
	yjfN	0.6	5.9	8.2	9.6	9.2	2.1
	chaA	2.2	6.4	12.1	9.5	11.7	4.2
	yhdV	1.3	1.9	5.6	9.2	9.1	12.8
	sdaA	1.4	4.1	9.1	9.2	18.2	6.7
	yodA	0.8	1.9	3.9	9.2	34.6	15.3
	mmuP	1.4	1.3	3.5	8.5	1.8	1.3
	metE	1.4	1.1	1.9	8.1	2.1	0.5
	kdpC	2.8	1.4	1.3	8.0	4.6	2.8
	frmB	1.4	1.5	3.1	7.9	17.8	9.7
	yobB	3.1	8.2	9.6	7.9	6.2	4.0

Values shown are fold changes relative to the pre-CORM culture sampled at the time points shown.

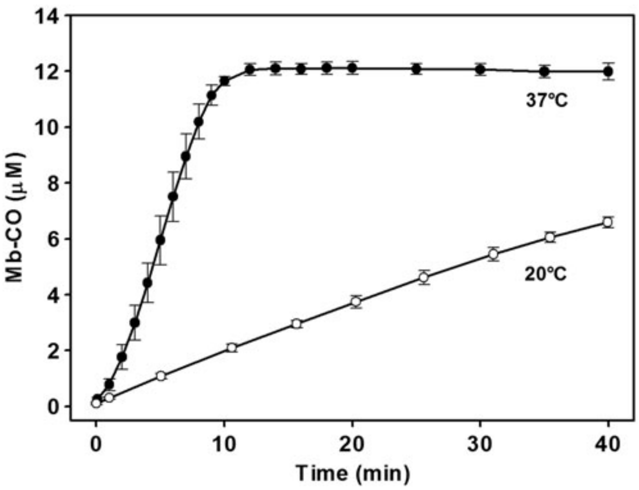

SUPPLEMENTARY FIG. S1. CO release from CORM-401 in 0.1 *M* KPi (pH 7.4) at 37°C or 20°C. Assays were performed in the presence of excess dithionite with myoglobin (20 μM) and 5 μM CORM-401. Under these conditions, CORM-401 releases 2.4 mole equivalent of CO with a $t_{1/2}$ of 5 min. $n = 3 \pm$ SEM.

SUPPLEMENTARY FIG. S2. TFInfer correlation profiles (coherence plots) showing TFs involved in the response to CORM-401 *versus* CO gas in *E. coli* cells. The *x*-coordinate of each point is presented with error bars and represents the profile difference between CORM-401 and CO treatments, computed as 1 minus the absolute Pearson correlation coefficient between the two profiles; the *y*-coordinate represents the change in magnitude of the response, computed as the difference of the norm of the two profiles. Data from aerobic (**A**, *top*) and anaerobic (**B**, *bottom*) conditions are shown. TFs whose response is similar for both CORM-401 and CO gas, both in magnitude and kinetics, will be located near the origin of each coherence plot in quadrant c, while TFs in quadrant b of each plot respond differently in both kinetics and amplitude. For example, CpxR, both aerobically and anaerobically, has a similar response in terms of the shape of the profile, but different magnitudes in (**A**) and (**B**), while ArcA (**A**) and ArgR (**B**) show similar magnitudes, but major differences in response profiles. TF, transcription factor.

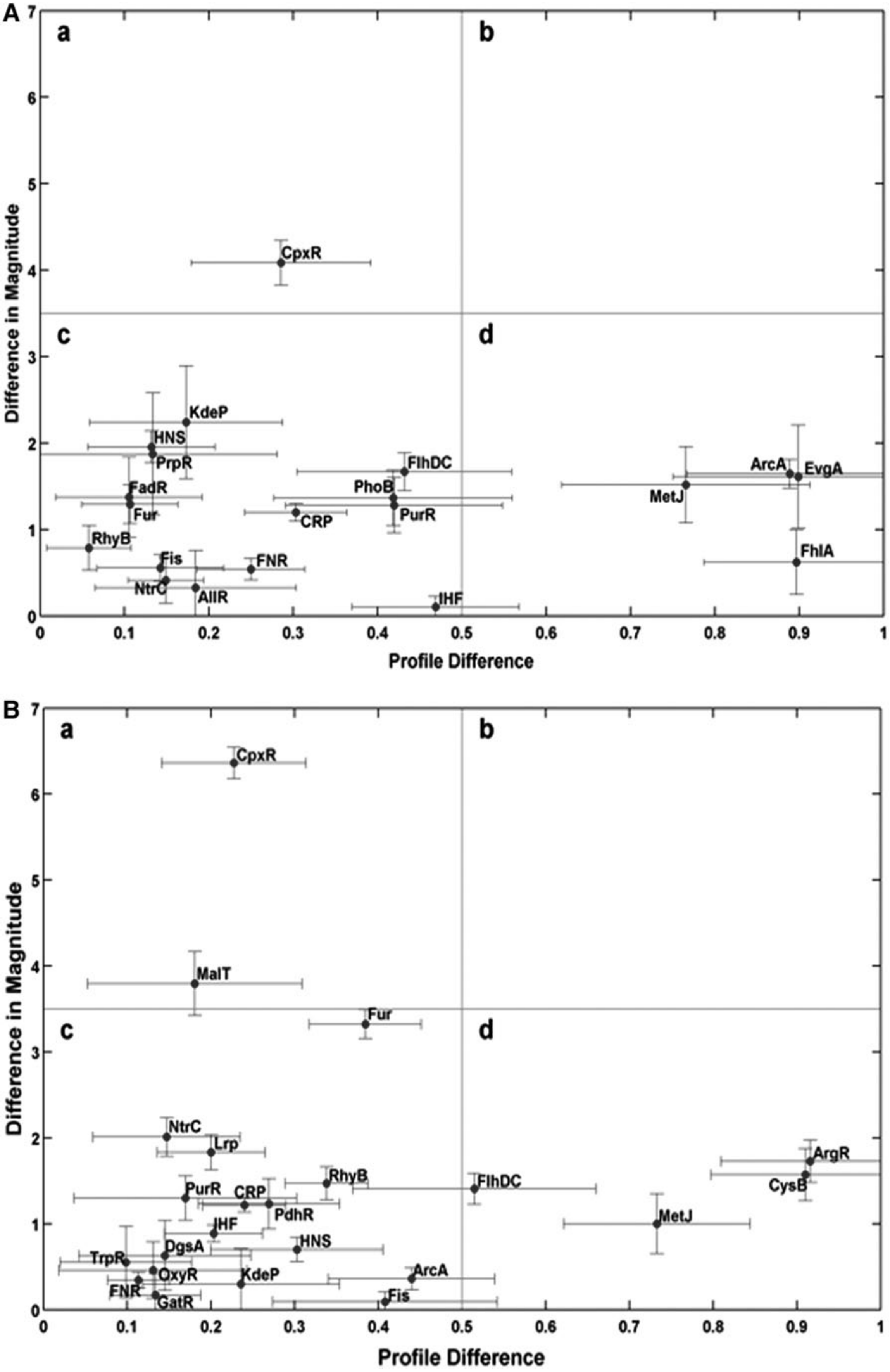
A
a
b
c
d
Difference in Magnitude
Profile Difference
CpxR
KdeP
HNS
PrpR
FadR
Fur
RhyB
Fis
NtrC
AllR
FNR
CRP
PhoB
PurR
FlhDC
IHF
MetJ
ArcA
EvgA
FhlA
B
a
b
c
d
Difference in Magnitude
Profile Difference
CpxR
MalT
Fur
NtrC
Lrp
RhyB
FlhDC
PurR
CRP
PdhR
IHF
HNS
DgsA
TrpR
OxyR
KdeP
ArcA
FNR
GatR
Fis
MetJ
ArgR
CysB

A

Fold change down-regulated						Fold change up-regulated				
30+	20-30	10-20	5-10	2-5	0.0	2-5	5-10	10-20	20-30	30+

Gene	Function	Relevant regulatory proteins	CORM-401 AEROBIC Time 2.5	5.0	10.0	20.0	40.0	80.0	CORM-401 ANAEROBIC Time 2.5	5.0	10.0	20.0	40.0	80.0
cyoA	cytochrome *bo'* terminal oxidase subunit II	ArcA (-)			0.44	0.20			0.44	0.11	0.09	0.20		
cyoB	cytochrome *bo'* terminal oxidase subunit I	ArcA (-)				0.23			0.50	0.33	0.32	0.38		
cyoC	cytochrome *bo'* terminal oxidase subunit III	ArcA (-)				0.20				0.22	0.26	0.34		
cyoD	cytochrome *bo'* terminal oxidase subunit IV	ArcA (-)				0.20				0.23	0.21	0.26	0.48	
cyoE	heme *o* synthase	ArcA (-)				0.21				0.33	0.36	0.37	0.48	
cydA	cytochrome *bd-I* terminal oxidase subunit I	ArcA (+), FNR (-)		2.88	2.42		2.86	3.07	2.01		0.32	0.26	0.25	0.38
cydB	cytochrome *bd-I* terminal oxidase subunit II	ArcA (+), FNR (-)		2.54	2.37	2.07	2.83	2.70			0.42	0.30	0.22	0.39
nuoA	NADH:ubiquinone oxidoreductase I	ArcA (-), FNR (-)		0.49		0.43					0.39	0.29	0.27	0.39
nuoB	NADH:ubiquinone oxidoreductase I	ArcA (-), FNR (-)										0.43	0.39	
nuoC	NADH:ubiquinone oxidoreductase I	ArcA (-), FNR (-)				0.49					0.46	0.33	0.35	
nuoE	NADH:ubiquinone oxidoreductase I	ArcA (-), FNR (-)				0.44					0.45	0.33	0.34	
nuoF	NADH:ubiquinone oxidoreductase I	ArcA (-), FNR (-)									0.47	0.34	0.31	
nuoG	NADH:ubiquinone oxidoreductase I	ArcA (-), FNR (-)									0.50	0.32	0.26	0.48
nuoH	NADH:ubiquinone oxidoreductase I	ArcA (-), FNR (-)										0.41	0.30	
nuoI	NADH:ubiquinone oxidoreductase I	ArcA (-), FNR (-)										0.43	0.32	
nuoJ	NADH:ubiquinone oxidoreductase I	ArcA (-), FNR (-)										0.47	0.34	
nuoK	NADH:ubiquinone oxidoreductase I	ArcA (-), FNR (-)										0.39	0.31	
nuoL	NADH:ubiquinone oxidoreductase I	ArcA (-), FNR (-)										0.47	0.35	
nuoM	NADH:ubiquinone oxidoreductase I	ArcA (-), FNR (-)										0.35	0.28	0.48
nuoN	NADH:ubiquinone oxidoreductase I	ArcA (-), FNR (-)											0.36	
ndh	NADH dehydrogenase II	FNR (-)			2.27	2.50	2.45	2.04						

B

Fold change down-regulated						Fold change up-regulated				
30+	20-30	10-20	5-10	2-5	0.0	2-5	5-10	10-20	20-30	30+

Gene	Product/Function	Relevant regulatory proteins	CORM-401 AEROBIC Time 2.5	5.0	10.0	20.0	40.0	80.0	CORM-401 ANAEROBIC Time 2.5	5.0	10.0	20.0	40.0	80.0
chaA	Na+ : K+/H+ antiporter		2.2	7.0	14.4	9.8	5.8	4.1	2.2	6.4	12.1	9.5	11.7	4.2
kdpA	K+ transporting ATPase subunit	KdpE (+)		2.9	11.3	15.6						2.1	2.3	
kdpB	K+ transporting ATPase subunit	KdpE (+)			9.8	13.8	7.5	4.8				3.7	3.4	2.0
kdpC	K+ transporting ATPase subunit	KdpE (+)	0.4		32.8	33.8	18.2	5.5	2.8				4.6	2.8
kdpD	regulator of the K+ transporting ATPase						2.1					2.5		
kdpE	regulator of the K+ transporting ATPase	KdpD-P (+)					2.7	2.6	2.0			2.4	2.3	2.0
kdpF	K+ transporting ATPase subunit			6.2	20.8	28.0		2.0		0.5				
kefA (mscK)	Potassium-dependent mechanosensitive channel					2.5	2.6					2.0		
kefB	K+ : H+ antiporter			0.3	0.1	0.1								
kefC	K+ : H+ antiporter													
kefF	Regulator of the Kef transporter					0.2								
kefG	Protein required for KefB activity				0.4	0.5								
trkA	K+ transporter			2.3	2.6									
znuA	Zn2+ ABC transporter	Zur (-)		2.6	6.5	11.2	10.0	5.2			3.4	4.3	7.2	6.4
mscL	mechanosensitive channel	RpoS (+)				0.4	0.4							
mscS	mechanosensitive channel	RpoS (+)		2.9	4.7	3.9					2.3	4.6	3.8	2.6
aqpZ	Aquaporin				0.3	0.4	0.4							
ompC	Outer membrane porin C	CpxR (-), OmpR (-)			2.1		3.1	2.5						
ompF	Outer membrane porin F	CpxR (-), OmpR (-)	0.5	0.2	0.2	0.0	0.1	0.2			0.2	0.0	0.0	0.0

C

Fold change down-regulated						Fold change up-regulated				
30+	20-30	10-20	5-10	2-5	0.0	2-5	5-10	10-20	20-30	30+

Gene	Product/Function	Relevant regulatory proteins	CORM-401 AEROBIC CONDITIONS Time 2.5	5.0	10.0	20.0	40.0	80.0	CORM-401 ANAEROBIC CONDITIONS Time 2.5	5.0	10.0	20.0	40.0	80.0
baeR	DNA-binding response regulator	CpxR (+), BaeR (+)			4.2	5.9	6.6	4.3			3.7	5.3	4.8	3.5
baeS	Sensory histidine kinase	CpxR (+), BaeR (+)		2.3	25.1	45.4	67.4	19.7			31.0	38.9	25.2	14.3
acrD	AcrAD-TolC multidrug efflux system; permease subunit	CpxR (+), BaeR (+)	4.8	10.7	15.2	11.2	10.9	6.9		8.9	11.3	11.1	8.2	7.6
marA	DNA-binding transcriptional dual regulator	CpxR (+)	3.0	3.3	4.9	4.7	7.8	2.7	3.6	11.4	16.7	28.6	47.8	20.4
marB	Polypeptide: multiple antibiotic resistance protein	CpxR (+)		2.2		4.4	6.5		3.3	5.6	13.9	21.7	26.7	10.3
marR	DNA-binding transcriptional repressor	CpxR (+)				2.5	3.2			5.1	8.3	10.9	20.2	10.8
mdtA	Multidrug efflux system; subunit A	CpxR (+), BaeR (+)	12.4	29.6	46.3	63.2	41.8	17.1	12.4	35.2	63.0	59.9	60.2	39.3
mdtB	Multidrug efflux system; subunit B	CpxR (+), BaeR (+)	4.4	24.3	32.6	42.5	30.2	10.8		10.4	24.8	23.0	20.8	18.4
mdtC	Multidrug efflux system; subunit C	CpxR (+), BaeR (+)		9.8	24.7	22.5	24.2	8.6		7.9	22.4	29.8	21.5	17.0
mdtD	Predicted transport protein	CpxR (+), BaeR (+)				2.0					2.6	2.7	2.4	2.3
spy	Envelope stress protein	CpxR (+), BaeR (+)	34.3	124.4	357.1	330.3	420.8	250.2	14.3	200.4	585.0	505.5	672.6	556.4
slt	Soluble lytic murein transglycosylase	CpxR (+)		2.5	3.8	3.4	3.5	2.5		2.5	3.8	3.4	3.5	2.5
degP	Serine protease	CpxR (+)	2.2			7.5	7.4	4.1		2.0	18.9	18.5	23.9	12.0
cpxA	Sensory histidine kinase	CpxR (+)				2.0				2.0	2.9	2.8	2.1	2.5
cpxP	Inhibitor of the Cpx response	CpxR (+)	12.3	17.2	17.7	13.6	16.8	10.6	4.6	11.9	17.0	12.9	11.5	5.9
cpxR	Transcriptional dual regulator	CpxR (+)		2.3	3.4	3.2	2.6	2.0		3.1	3.4	3.4	3.3	3.1

SUPPLEMENTARY FIG. S3. Differential expression of genes, both aerobically and anaerobically, in response to 67 μ*M* CORM-401, involved in (A) the respiratory chains, (B) osmoregulation, and (C) general stress responses, metal ion stress, and cell envelope stress. Values within each cell are fold changes in transcript levels relative to the no-CORM condition. The heat map (*above*) also quantifies the changes elicited in selected genes.

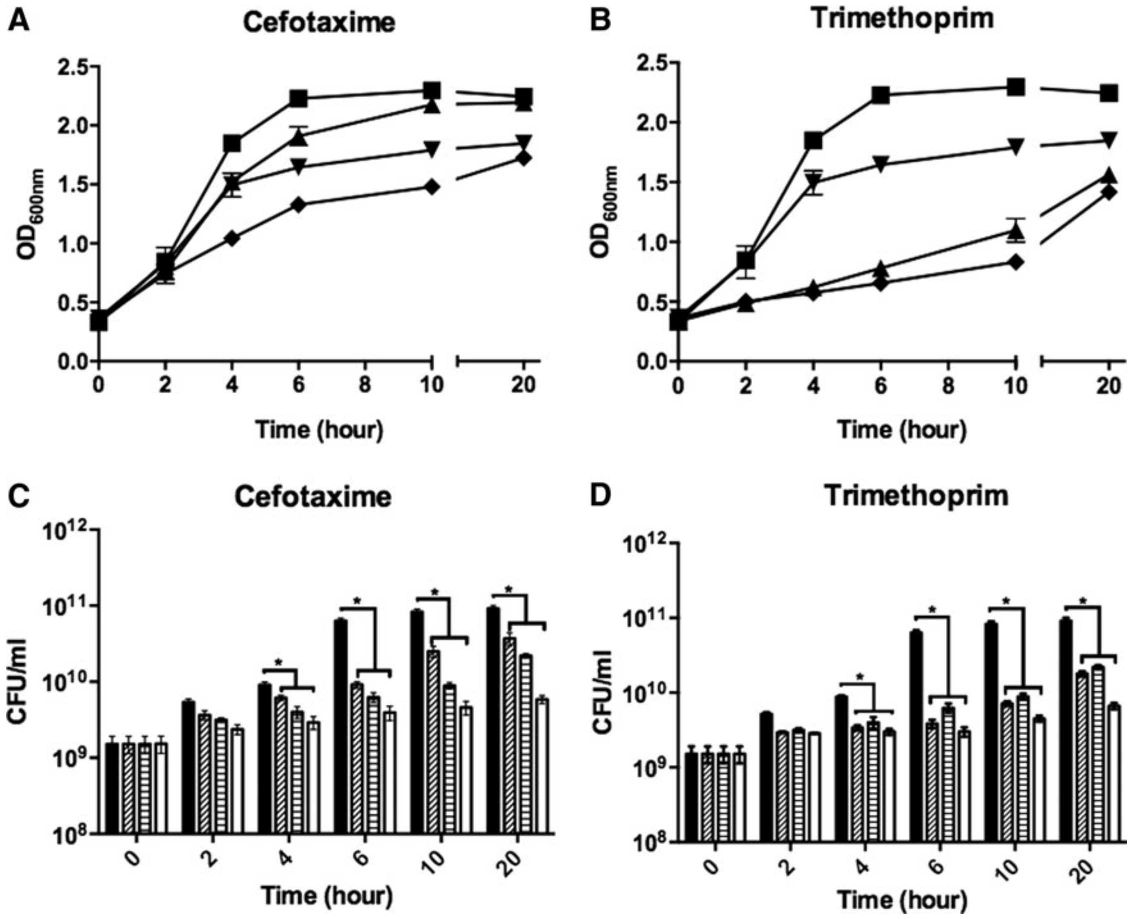

SUPPLEMENTARY FIG. S4. CORM-401 enhances the antimicrobial effects of cefotaxime and trimethoprim with additive effects on growth and viability. In **(A)** and **(B)**, bacterial growth was followed in Evans medium with glucose until OD_{600nm} ~0.3 was reached. CORM-401 and antibiotics were then added to the cultures; OD readings and samples for viability were taken at regular intervals. In **(A)** (cefotaxime) and **(B)** (trimethoprim), *squares* show control cultures, *triangles* are antibiotic alone (1 μg ml^{-1}), *inverted triangles* are CORM-401 alone (100 μM), and *diamonds* are antibiotic and CORM combined. In **(C)** (cefotaxime) and **(D)** (trimethoprim), *black columns* show control cultures, *diagonal hatched bars* are antibiotic alone (1 μg ml^{-1}), *horizontally striped bars* are CORM-401 alone (100 μM), and *open bars* are antibiotic and CORM combined. $n = 3 \pm$ SD. *, $p < 0.05$ (T-test).
